# Cantonese Tone Identification in Three Temporal Cues in Quiet, Speech-Shaped Noise and Two-Talker Babble

**DOI:** 10.3389/fpsyg.2018.01604

**Published:** 2018-10-09

**Authors:** Puisan Wong, Sheung Ting Cheng, Fei Chen

**Affiliations:** ^1^Division of Speech and Hearing Sciences, Faculty of Education, University of Hong Kong, Hong Kong, Hong Kong; ^2^Department of Electrical and Electronic Engineering, Southern University of Science and Technology, Shenzhen, China

**Keywords:** temporal cue, Cantonese tone, cochlear implant, temporal envelope, temporal fine structure, periodicity, tone perception, perception in noise

## Abstract

**Purpose:** Cochlear implant processors deliver mostly temporal envelope information and limited fundamental frequency (F0) information to the users, which make pitch and lexical tone perception challenging for cochlear implantees. Different factors have been found to affect Mandarin tone perception in temporal cues but the most effective temporal cues for lexical tone identification across different backgrounds remained unclear because no study has comprehensively examined the effects and interactions of these factors, particularly, in languages that use both pitch heights and pitch shapes to differentiate lexical meanings. The present study compared identification of Cantonese tones in naturally produced stimuli, and in three temporal cues, namely the amplitude contour cue (TE50), the periodicity cue (TE500), and the temporal fine structure cue (TFS), in three different numbers of frequency bands (B04, B08, B16) in quiet and two types of noise (two male talker-babble and speech-shaped noise).

**Method:** Naturally produced Cantonese tones and synthetic tones that combined different acoustic cues and different number of frequency bands were presented to 18 young native Cantonese speakers for tone identification in quiet and noise.

**Results:** Among the three temporal cues, TFS was the most effective for Cantonese tone identification in quiet and noise, except for T4 (LF) identification. Its effect was even stronger when the tones were presented in 4 or 8 bands rather than 16 bands. Neither TE500 nor TE50 was effective for Cantonese tone identification in quiet or noise. In noise, most tones in TE500 and TE50 were misheard as T4 (LF), demonstrating errors in both tone shapes and tone heights. Types of noise had limited effect on tone identification.

**Conclusions:** Findings on Mandarin tone perception in temporal cues may not be applicable to other tone languages with more complex tonal systems. TFS presented in four bands was the most effective temporal cue for Cantonese tone identification in quiet and noise. Temporal envelope cues were not effective for tone, tone shape or tone height identification in Cantonese. These findings have implications for future design of cochlear implants for tone speakers who use pitch heights or a combination of pitch heights and pitch shapes to differentiate meanings.

## Introduction

Pitch is used in languages to serve pragmatic, emotional and linguistic purposes (Barry et al., 2002; Plag et al., 2011). Pitch is also used to segregate speech from noise and voices of different speakers when listening to speech in noise (Oxenham, 2008). In tonal languages, pitch plays an extra role in making semantic contrasts. Syllables produced with different pitch shapes and/or pitch heights convey different meanings (Barry et al., 2002).

Pitch perception is difficult for many populations. Older listeners with near-normal hearing have poorer than normal pitch perception (Moore and Peters, 1992). Hearing-impaired listeners with poorer frequency selectivity may experience poorer complex pitch perception (Oxenham, 2008). Even normal-hearing listeners have poorer pitch perception in the presence of background noise, particularly when the signal-to-noise ratio (SNR) was at or lower than −10 dB (Kong and Zeng, 2006).

Cochlear implant users have extra difficulties perceiving pitch because current speech-processing strategies for cochlear implants deliver primarily the temporal information in speech signals but not the spectral information such as the fundamental frequency (F0) (Kong and Zeng, 2006). Because tonal languages use pitch to contrast lexical items, cochlear implant users speaking a tone language encounter even greater difficulties in speech processing than cochlear implant users speaking a non-tonal language due to the lack of pitch information for recognizing lexical meanings (Barry et al., 2002).

Although F0 is the primary cue for pitch and tone recognition, several studies have shown that temporal cues also contribute significantly to pitch perception. Rosen (1992) identified three temporal cues, which included two temporal envelope cues and one temporal fine structure cue, for speech perception. The two temporal envelope cues were the amplitude contour cue and the periodicity cue which referred to amplitude fluctuation in the speech signal between 2 and 50 Hz and between 50-500 Hz, respectively. The temporal fine structure cue was the high-frequency fluctuation between 500-10,000 Hz (Rosen, 1992). How effective these temporal cues are for lexical identification is unclear.

Studies that examined the importance of the two temporal envelope cues for Mandarin tone perception in quiet found inconsistent results. Fu and Zeng (2000) and Kuo et al. (2008) explored the relative importance of the amplitude contour, periodicity and duration cues in Mandarin tone recognition and found that the amplitude contour cue and the periodicity cue contributed to tone recognition when F0 was not available, whereas the duration cue only played a minor role. Fu et al. (1998) studied the effectiveness of the amplitude contour cue and periodicity cue in Mandarin tone recognition using half-wave rectification and low-pass filtering of the speech signal at 50 Hz and 500 Hz, respectively. They reported that tone recognition with either cue was well above chance. When the low-pass cutoff frequency of the envelope extraction filters was increased from 50 to 500 Hz, Mandarin tone recognition improved from 67 to 81%, suggesting that the periodicity cue was more effective than the amplitude envelop cue for Mandarin tone perception. On the other hand, Fu and Zeng (2000) reported that identification accuracies of the four Mandarin tones were 45, 36, 77, and 78% (mean = 59%) using the amplitude contour cue and 56, 46, 50, and 64% (mean = 54%) using the periodicity cue. These findings contradicted those in Fu et al. (1998). Tone identification accuracies in the two temporal envelope cues were lower in this study than in Fu et al. (1998). Also, there was a lack of an advantage of the periodicity cue over the amplitude envelope cue for Mandarin tone identification in Fu and Zeng (2000). The findings also suggested that tone accuracies using temporal envelope cues were affected by tone shapes. T4 (HF), the high falling tone in Mandarin was identified with high accuracies than the other three tones in both temporal envelope cues.

Two studies examined the effect of number of frequency bands (i.e., amount of spectral information) on Mandarin tone identification in the two temporal envelope cues in quiet and reported mixed results. Fu et al. (1998) divided speech into one to four broad bands and extracted the amplitude contour cue and periodicity cue of each band. The extracted temporal envelope cues were used to modulate broadband noises and then combined to form signals with amplitude contour cues and periodicity cues in one to four frequency bands. Signals that were combined from more frequency bands provided more spectral information. Native Mandarin speakers were asked to identify the tones in quiet to determine the effect of frequency bands. The results demonstrated no effect on tone recognition in the two temporal envelope cues with an increase in the spectral information from 1 to 4 frequency bands. Nevertheless, Xu et al. (2002) found an interaction effect of number of frequency bands and types of temporal envelope cues when the number of frequency bands increased from 1 to 12. Tones presented in amplitude envelope cues with more frequency bands (i.e., less detailed temporal envelope cue but more spectral information) were perceived with similar accuracy as tones presented in periodicity cues with fewer frequency bands (i.e., more detailed envelope cue but less spectral information).

Temporal fine structure appeared to be a much more effective cue for Mandarin tone perception than amplitude envelop in quiet situations. Xu and Pfingst (2003) used synthetic stimuli which combined the amplitude envelope of one tone and the temporal fine structure of another (known as “auditory chimera”). Listeners identified the tones in quiet. The results showed that over 80% of participants' responses were consistent with the tones presented in the temporal fine structure. The authors concluded that temporal fine structure was a much stronger acoustic cue for Mandarin tone identification than the amplitude envelope cue in a quiet condition.

Very few studies have examined tone identification in temporal cues in noise. Kong and Zeng (2006) found that periodicity and amplitude contour cues were more vulnerable to noise than the temporal fine structure cue, suggesting that temporal envelope cues are less salient for tone perception in noise. Unlike previous studies that extracted the amplitude modulations in the speech signal to generate the temporal fine structure cue using Hilbert transformation, this study combined amplitude and frequency modulations to create the temporal fine structure cue. The authors further observed an interaction effect of the envelope cues and number of frequency bands for tone recognition in quiet and noise. Under the quiet condition, tone identification with the periodicity cue in one band was better than tone identification with the amplitude envelope cue in 8 bands. Yet this pattern was reversed in the noise condition, indicating that the effect of the temporal envelope cues under the noise condition was affected by the amount of spectral information in the signal.

Different types of noise induce different amounts of energetic and informational masking (Mattys et al., 2009) and may influence tone recognition differently. For example, speech-shaped noise imposes mostly energetic masking (Lecumberri and Cooke, 2006) while noise babbles with two talkers in the background yield the most informational masking (Freyman et al., 2004). Energetic masking leads to signal degradation while informational masking leads to depletion of high-order processing resources (Mattys et al., 2009). Yet most previous studies only used speech-shaped noise to examine the effects of noise on tone perception and did not systematically examine the effect of noise types on tone recognition. This study compared tone perception in a two talker-babble and speech-shaped noise to examine the effect of energetic and informational masking on tone perception in temporal cues.

A majority of studies that examined tone recognition in different temporal cues focused on Mandarin, which has a relatively simple tonal system in which each tone has a unique pitch contour shape. Though findings from these studies provide information on the identification of tones in a tone system that contrasts meanings with tone shapes, it is unclear whether the results are applicable to tone perception in tone languages which use pitch heights (e.g., many African languages) or both pitch heights and pitch shapes (e.g., Thai and Cantonese) to contrast lexical items.

Cantonese is particularly well suited for examining tone perception in different acoustic cues. It has one of the most complex tonal systems that is contrastive in both tone shapes and tone heights (see Wong and Chan (2018) for a detailed description of the acoustic properties of Cantonese tones). Cantonese has three level tones [i.e., Tone 1 (T1, High Level, HL), Tone 3 (T3, Mid-Level, ML), and Tone 6 (T6, Low Level, LL)], two rising tones [i.e., Tone 2 (T2, High Rising, HR), Tone 5 (T5, Low Rising, LR)], and one falling tone [i.e., Tone 4 (T4, Low Falling, LF)]. These tones are contrasted by pitch height, pitch shapes or both. For examples, the three level tones [T1 (HL), T3 (ML) and T6 (LL)] are contrasted by tone height. T2 (HR)–T5 (LR), and T4 (LF)–T5 (LR) are differentiated by tone shape, the former pair differing by the slope of the rising pitch and latter differing by the direction of pitch change. T1 (HL)–T5 (LR) differ by both tone height and tone shape (see Figure 2 in Wong and Chan, 2018 for the pitch contours of the tones). Both children (Wong et al., 2017; Wong and Leung, 2018) and adults have difficulty identifying Cantonese tones with similar shapes but different pitch levels or pitch slopes (e.g., T3 ML vs. T6 LL, and T2 HR vs. T5 LR) (Wong and Chan, 2018; Wong and Ng, 2018). Thus, examining Cantonese tone perception in different temporal cues would provide insights into tone identification in languages that use pitch levels only, pitch shapes only or both to convey meaning. It would also have implications for music perception which involves perception of pitch levels and pitch shapes.

Only two studies have examined Cantonese tone perception in different temporal cues. Yuen et al. (2007) mainly compared Cantonese tone identification in quiet using the periodicity cue extracted from different frequency ranges and reported that higher frequency bands were more important for Cantonese tone identification than lower frequency bands. Yuan et al. (2009) studied the contribution of the periodicity cue and an enhanced periodicity cue in Cantonese tone and word identification in quiet and in speech-shaped noise using full-wave rectification and low-pass filtering. Listeners were asked to identify the words they heard by selecting one of four disyllabic words presented on the screen. The four words differed in 2 tones in the one of the syllables (e.g., do1 jyu1 “more than,” do1 jyu4 “in excess,” do1 syu1 “lots of books,” do1 ji4, “suspicious”). The results showed that tone identification with the periodicity cue could reach a high performance level in quiet but deteriorated in noise.

These studies revealed that various factors, such as type of temporal cues, number of frequency bands, and speech-shaped noise that generates energetic masking, affected lexical tone perception. However, due to the inconsistent findings, the lack of studies that comprehensively examined the interactions of all these factors in the same group of listeners, and the lack of adoption of noise that created informational masking, it remains unclear which temporal cue would be the most effective for lexical tone perception across quiet and different noisy situations. In addition, whether the findings from Mandarin studies can be applied to other tone languages is also unclear. Thus, no confident conclusion can be drawn about whether the acoustic cues delivered through current cochlear implants are sufficient or the most effective for lexical tone identification in different backgrounds and in tone languages with tone height and/or tone shape contrasts.

This study aimed to investigate the effect of the three temporal cues—the amplitude contour cue, the periodicity cue, and the temporal fine structure cue—on the perception of Cantonese tones in different number of frequency bands in quiet and in two types of noise. The specific research questions were:

1. How well do listeners identify the six Cantonese tones in naturally produced speech stimuli in quiet and in noise?
2. Which temporal cues are more effective for Cantonese tone identification in quiet and in different types of noise?
3. How well are tone heights and tone shapes identified in different temporal cues in quiet and noise?

Based on the fact that different types of noise produce different masking effects, it was predicted that tone perception accuracies in different temporal cues would differ in the two types of noise, namely speech shape noise, which mostly generates energetic masking, and two-talker babble, which mostly induce informational masking. Based on the findings of Fu and Zeng (2000) and Fu et al. (1998) on Mandarin tones, we expected that in quiet condition, the two temporal envelope cues (i.e., TE500 and TE50) would be effective for tone identification, though not as good as the TFS cue (Xu and Pfingst, 2003). Among the different tone shapes, the falling shape would be the easiest to identify in TE500 and TE50 (Fu et al., 1998). If the findings of Fu et al. (1998) held up for Cantonese tones, there would not be an interaction between number of bands and the two temporal cues. However, if the findings of Xu et al. (2002) were applicable, there would be an interaction between number of frequency bands and the two temporal envelope cues, which meant that tone identification in TE500 would be better in fewer frequency bands, whereas identification accuracy in TE50 would be higher with more frequency bands. When the tones were presented in noise, tone and tone shape identification accuracy in the two temporal envelope cues, but not in the TFS cue, would drop significantly (Kong and Zeng, 2006). Given that native speakers have difficulties differentiating tones differ by tone heights in quiet, it was also predicted that identification of tone heights in temporal cues would be more difficult than the identification of tone shapes.

## Methods

The protocol used in this study was approved by the Human Research Ethics Committee at the University of Hong Kong (EA530114).

### Participants

Twenty native young Cantonese speakers, 10 male and 10 female, between 19 and 24 years of age (mean age = 21.5 years) without speech and language disability were recruited. Half of them were studying Speech and Hearing Sciences at The University of Hong Kong. Cantonese was reported to be their first, dominant and home language. No participants knew any other tonal language or Chinese dialect, but had limited exposure to Mandarin in school. All passed a hearing screening at 25 dB HL at octave frequencies between 500 and 8,000 Hz bilaterally.

Because Cantonese is a spoken language and tones are not specified in writing, many Cantonese speakers without phonetic training lack the metalinguistic skill to accurately label Cantonese tones, particularly the acoustically similar tones (i.e., T3 (ML)–T6 (LL), T2 (HR)–T5 (LR), and T4 (LF) and T6 (LL)). Also, some native Cantonese speakers merge these tone categories in their perception and production (Bauer et al., 2003; see Wong and Chan, 2018 for a review). To ensure that participants could identify and produce the six Cantonese tones accurately, only participants who were able to correctly repeat real and non-sense words of the six tones in a phone screening test were included. Two of the 20 participants who failed to identify T6 (LL) in the original signal in any of the trials in quiet were excluded. As a result, data from the remaining 18 participants were analyzed.

### Stimuli

#### Original natural speech stimuli

Eighteen familiar Cantonese monosyllabic words (3 syllables × 6 lexical tones) were recorded by a male native Cantonese-speaker and used as the experimental stimuli (see Table 1 for the word list). Six familiar Cantonese monosyllabic words (1 syllable × 6 lexical tones) were recorded by a female speaker and were used for training. All stimuli were recorded in a soundproof room with a Shure SM58 microphone at a sampling rate of 44.1 kHz.

**Table 1:** Experimental stimuli.

**Monosyllabic target in IPA**	**Chinese character**	**English meaning**
/fu1/	夫	Man
/fu2/	苦	Bitter
/fu3/	富	Rich
/fu4/	符	Symbol
/fu5/	婦	Woman
/fu6/	負	Carry
/ji1/	衣	Clothes
/ji2/	椅	Chair
/ji3/	意	Meaning
/ji4/	兒	Son
/ji5/	已	Finished
/ji6/	二	Two
/jyun1/	冤	Injustice
/jyun2/	丸	Pill
/jyun3/	怨	Hatred
/jyun4/	圓	Round
/jyun5/	遠	Far
/jyun6/	願	Wish

*IPA stands for international phonetic alphabet*.

#### Stimuli of different temporal cues

Each set of stimuli was generated using the noise-excited vocoder method similar to that in Shannon et al. (1995). Because most recent versions of cochlear implant processors employ a larger number of frequency bands (between 8 and 20 bands; Loizou, 1998), 4, 8, and 16 frequency bands were adopted in the experimental conditions to simulate the spectral information available in current implant systems. Another set of 32 frequency bands was adopted in the training condition to avoid an unbalanced learning effect in the experimental conditions.

To construct the stimuli, the original speech stimuli were first passed through a pre-emphasis filter and divided into 1, 4, 8, 16, and 32 frequency bands using a bandpass analysis filter whose cut-off frequencies spaced the cochlear frequency map with equal steps, and were computed according to the cochlear frequency-position mapping function (Greenwood, 1990). A Hilbert transform was then used to extract the temporal envelope in each analysis band followed by low-pass filtering at either 50 or 500 Hz to obtain the amplitude envelope cue (TE50) and the periodicity cue (TE500), respectively. The temporal envelope cues were then used to modulate a white noise, which was then bandpass filtered by the same analysis filters used in the original analysis band. These envelope-modulated narrow-band signals were then resynthesized to create the final stimuli. The speech energy was normalized. For the temporal fine structure cue (TFS), the original stimuli were processed using the Hilbert transformation, described above, to extract the high-frequency fluctuations.

#### Noises and combination of signal and noise

Three types of noise including a two male talker-babble (2MB), a two female talker-babble (2FB) and speech-shaped noise (SSN) were used for the noise conditions for training and experimental conditions. Five male and five female native Cantonese speakers were invited to record 25 daily sentences, selected from the Cantonese Hearing in Noise Test (CHINT) (Wong and Soli, 2005), which included all the phonemes of Cantonese, in a sound-treated booth. Each sentence was repeated four times and a native Cantonese speaker selected the most natural and clear recording of each sentence by each speaker. Two male speakers were randomly selected and their 25 sentences were normalized to the same RMS level and connected in a random order. The production of the two speakers were then added together to create the 2MB. The 2FB for the training stimuli was created using the same procedures on the productions of two randomly selected female speakers. The SSN was generated by passing white noise through a 125-coefficient filter derived from the Linear Predictive Coding spectrum of the 250 sentences of all the 10 speakers.

The original and temporal cue stimuli were equalized in RMS energy. The stimuli were combined with a randomly selected portion of the two noises and the intensity level of the noises was adjusted to form 0 dB SNR with the speech stimuli.

#### Final experimental and training stimuli

Altogether, 9 blocks, 3 frequency bands (i.e., B04, B08, B16) and 3 listening conditions (i.e., quiet, 2MB, and SSN) of experimental stimuli and 4 blocks of training stimuli were prepared. Each experimental block consisted of 72 stimuli: 3 syllables × 6 tones × 4 cues (i.e., original production, TE50, TE500, and TFS). For the training stimuli, the first block consisted of the six original training stimuli (1 syllable × 6 tones) in quiet that were filtered and re-synthesized from 32 frequency bands. The other three training blocks were constructed with 32 frequency bands and consisted of 18 stimuli: 1 syllable × 6 tones × 3 temporal cues (TE50, TF50, TFS). One block was presented in quiet, one in SSN and one in 2FB.

### Procedure

Participants were invited to attend a 2-h session in a double-walled sound-treated sound booth. They were first asked to complete a questionnaire about their language background and received a hearing screening at 25 dB HL at octave frequencies between 500 and 8,000 Hz.

After that, training was provided before the experiment. During training, a training block with the original stimuli in quiet was presented first, followed by a training block with the three cues in quiet. The other two training blocks, each with the three cues in one type of background noise (i.e., 2FB and SSN), were presented randomly and order was balanced across participants. No feedback was provided in the training blocks. Data collected in the training blocks were not used for analysis.

In the experiment, the stimuli were blocked by the number of frequency bands and the order of presentation was counterbalanced across subjects. Within each frequency band, the quiet condition was presented first. After that, participants listened to the stimuli blocked by noise types. The order of presentation of the noise types was randomly presented. Stimuli of different tones and different syllables in the original productions and in the three temporal cues acoustic cue were mixed together in each block. Trials within blocks were randomly presented.

In both the training and experimental blocks, stimuli were presented to listeners bilaterally at a fixed level of approximately 65 dBA through Sennheiser HD 380 Pro headphones without repetition. Six Chinese characters representing the syllable in the six tones were shown on the computer screen. Because participants might not be able to hear the segmental information of words in the temporal cues, and those who did not have phonetic training, might not be able to label the tones even though they were able to perceive and produce them, to ensure that the participants selected the answers that represented the tones they heard, they were asked to repeat the tones/words they heard. Their productions were audio-recorded using a Shure SM58 microphone and were rated again later for checking inter- and intra-judge reliability. A phonetically-trained experimenter, who was sitting in front of the screen with the participant but did not hear the stimuli, judged the tones of the words produced by the participant and selected the answer on the screen for the participant. No feedback was given. Participants were encouraged to guess the word if they were not sure and to skip the trial if they could not hear the word.

## Results

### Intra- and inter-judge reliability

To ensure that tone judgment was reliable, the experimenter and another phonetically-trained student listened to the recordings of five randomly selected participants (25% of the data). Cohen's Kappa was used to determine inter- and intra- judge reliability. Results showed almost perfect agreement for both intra-judge reliability, (κ = 0.89, *p* < 0.001) and inter-judge reliability (κ = 0.85, *p* < 0.001), indicating that the judges reached almost perfect reliable in their judgment of the tones produced by participants.

### Data analyses

**Figure 1:**
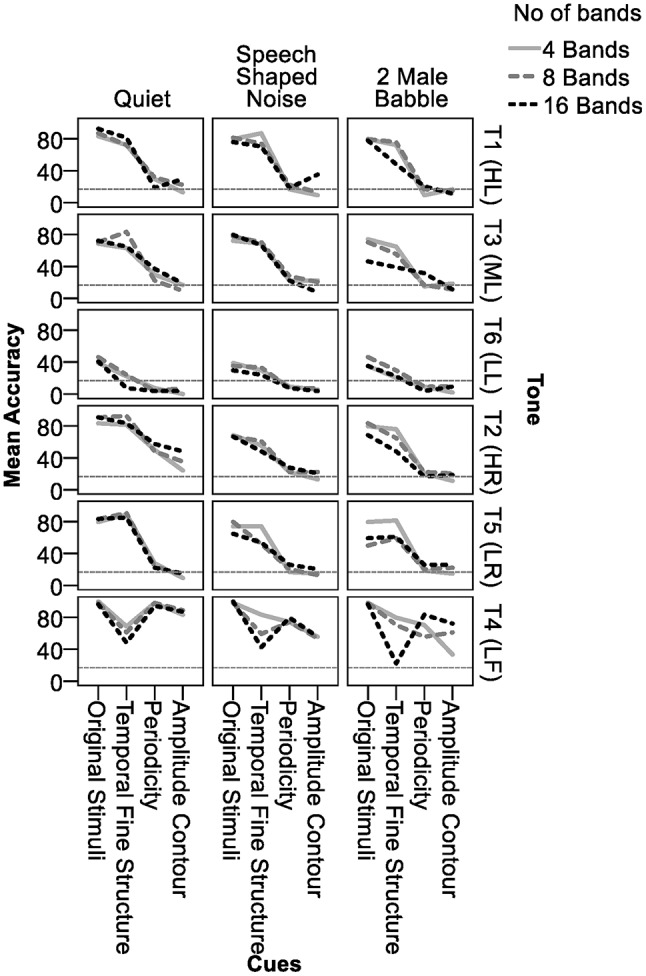
Tone accuracy in different temporal cues, number of frequency bands and backgrounds. The horizontal broken line indicates chance level.

Statistical analyses were based on the data collected from 18 participants. Data from participant 2 and 14 were excluded due to 0% accuracy in T6 (LL) identification in the original stimuli in quiet, indicating possible tone merge. Figure 1 shows the accuracy of tone identification in different conditions. The horizontal gray broken line marks chance level. To determine whether there were statistically significant differences and interactions in tone identification accuracies in different conditions, a four-way repeated measures analysis of variance (ANOVA) was conducted, using tone identification accuracies as the dependent variable and Backgrounds (Quiet, 2MB, SSN), number of Bands (B04, B08, B16), acoustic Cues (original, TFS, TE500, TE50), and tones (T1, T2, T3, T4, T5, T6) as independent variables (Table 2). Given the significant 4-way interactions and the significant 3- and 2-way interactions of Backgrounds with the other three factors (Table 2), a three-way repeated measures ANOVA was conducted for each type of Background (Table 3). The results showed that 3-way interactions of Cues × Bands × Tones was not significant in QUI and SSN, but was significant in 2MB (Table 3). Thus, a two-way Bands × Tones ANOVA was conducted for each of the four cues (Table 4) and a two-way Bands × Cues ANOVA was conducted for each of the six tones in 2MB (Table 5). Pairwise comparisons were then performed to determine the orders of Tone accuracies (Table 6), Band accuracies (Table 7), and Cue accuracies (Table 8) of different factors in different conditions in each type of backgrounds. To determine the effect of Backgrounds on different acoustic Cues, another three-way repeated measures ANOVA was conducted for each type of cues (Table 9). Given the significant three-way interactions of Backgrounds × Bands × Tones in TE50 (Table 9), a Backgrounds × Bands two-way ANOVA was performed for each tone for TE50 (Table 10). Pairwise comparisons were then conducted to determine the order of accuracy of Backgrounds for each Cue and Tone and Band (Table 11).

**Table 2:** Results of Backgrounds * Cues * Bands * Tones four-way repeated measures ANOVA on tone accuracy.

	**df1**	**df2**	***F***	***p***	**Partial η^2^**
**Backgrounds**	2.00	34.00	73.46	** <0.001**	0.81
**Cues**	1.50	25.50	433.30	** <0.001**	0.96
Bands	2.00	34.00	2.18	0.128	0.11
**Tones**	2.62	44.59	51.89	** <0.001**	0.75
Backgrounds * Cues	6.00	102.00	1.38	0.231	0.07
**Backgrounds** * **Bands**	4.00	68.00	2.88	**0.029**	0.14
**Backgrounds** * **Tones**	10.00	170.00	12.09	** <0.001**	0.42
**Cues** * **Bands**	6.00	102.00	18.97	** <0.001**	0.53
**Cues** * **Tones**	4.54	77.19	22.27	** <0.001**	0.57
Bands * Tones	10.00	170.00	1.16	0.320	0.06
**Backgrounds** * **Cues** * **Bands**	12.00	204.00	3.43	** <0.001**	0.17
Backgrounds * Bands * Tones	8.03	136.45	1.22	0.294	0.07
**Cues** * **Bands** * **Tones**	10.31	175.33	3.17	**0.001**	0.16
**Backgrounds** * **Cues** * **Tones**	10.36	176.08	5.10	** <0.001**	0.23
**Backgrounds** * **Cues** * **Bands** * **Tones**	13.12	222.99	1.95	**0.026**	0.10

*Significant effects are marked with bold font*.

**Table 3:** Results of Cues * Bands * Tones three-way repeated measures ANOVA on tone accuracy.

**Background**		**df1**	**df2**	***F***	***P***	**Partial η^2^**
QUI	**Cues**	3.00	51.00	335.04	** < 0.001**	0.95
	Bands	2.00	34.00	1.20	0.312	0.07
	**Tones**	2.45	41.58	62.75	** < 0.001**	0.79
	**Cues** * **Bands**	6.00	102.00	3.89	**0.002**	0.19
	**Cues** * **Tones**	5.93	100.75	26.09	**<0.001**	0.61
	Bands * Tones	10.00	170.00	1.15	0.331	0.06
	Cues * Bands * Tones	10.86	184.62	1.21	0.287	0.07
SSN	**Cues**	3.00	51.00	253.26	**<0.001**	0.94
	Bands	1.44	24.41	1.14	0.319	0.06
	**Tones**	3.17	53.86	36.51	**<0.001**	0.68
	**Cues** * **Bands**	6.00	102.00	4.56	**<0.001**	0.21
	**Cues** * **Tones**	5.67	96.39	9.03	**<0.001**	0.35
	Bands * Tones	10.00	170.00	0.71	0.713	0.04
	Cues * Bands * Tones	10.54	179.12	1.84	0.053	0.10
2MB	**Cues**	1.59	27.06	204.22	**<0.001**	0.92
	**Bands**	2.00	34.00	5.52	**0.008**	0.25
	**Tones**	5.00	85.00	32.86	**<0.001**	0.66
	**Cues** * **Bands**	6.00	102.00	16.89	**<0.001**	0.50
	**Cues** * **Tones**	6.18	105.03	10.03	**<0.001**	0.37
	Bands * Tones	5.42	92.20	1.76	0.123	0.09
	**Cues** * **Bands** * **Tones**	10.37	176.26	3.78	**<0.001**	0.18

*Significant effects are marked with bold font*.

**Table 4:** Results of Bands *Tones two-way repeated measures ANOVA on tone accuracy by Cues when Backgrounds = 2MB.

**Background**	**Cue**		**df1**	**df2**	***F***	***p***	**Partial η^2^**
**2MB**	**ORI**	**Bands**	2.00	34.00	5.44	**0.009**	0.24
		**Tones**	2.92	49.61	19.30	**<0.001**	0.53
		Bands*Tones	5.39	91.63	3.34	**0.007**	0.16
	**TFS**	**Bands**	2.00	34.00	28.43	**<0.001**	0.63
		**Tones**	5.00	85.00	14.46	**<0.001**	0.46
		Bands*Tones	10.00	170.00	3.68	**<0.001**	0.18
	**TE500**	**Bands**	2.00	34.00	4.40	**0.020**	0.21
		**Tones**	5.00	85.00	33.58	**<0.001**	0.66
		Bands*Tones	10.00	170.00	2.21	**0.019**	0.11
	**TE50**	**Bands**	2.00	34.00	6.27	**0.005**	0.27
		**Tones**	3.12	52.97	17.03	**<0.001**	0.50
		Bands*Tones	10.00	170.00	3.16	**<0.001**	0.16

*Significant effects are marked with bold font*.

**Table 5:** Results of Bands * Cues two-way repeated measures ANOVA on tone accuracy by Tones when Backgrounds = 2MB.

**Backgrounds**	**Tones**		**df1**	**df2**	***F***	***p***	**Partial η^2^**
**2MB**	**T1**	Bands	2.00	34.00	2.44	0.102	0.13
		**Cues**	1.55	26.31	89.40	**<0.001**	0.84
		**Bands** * **Cues**	6.00	102.00	3.46	**0.004**	0.17
	**T2**	**Bands**	2.00	34.00	4.72	**0.016**	0.22
		**Cues**	1.81	30.73	56.48	**<0.001**	0.77
		**Bands** * **Cues**	6.00	102.00	2.29	**0.041**	0.12
	**T3**	Bands	2.00	34.00	3.08	0.059	0.15
		**Cues**	3.00	51.00	36.05	**<0.001**	0.68
		**Bands** * **Cues**	6.00	102.00	4.93	**<0.001**	0.22
	**T4**	Bands	2.00	34.00	0.29	0.753	0.02
		**Cues**	3.00	51.00	35.33	**<0.001**	0.68
		**Bands** * **Cues**	6.00	102.00	17.22	**<0.001**	0.50
	**T5**	Bands	2.00	34.00	2.17	0.130	0.11
		**Cues**	3.00	51.00	58.67	**<0.001**	0.78
		**Bands** * **Cues**	6.00	102.00	4.01	**0.001**	0.19
	**T6**	Bands	2.00	34.00	2.19	0.128	0.11
		**Cues**	1.99	33.88	18.57	**<0.001**	0.52
		Bands * Cues	6.00	102.00	0.60	0.732	0.03

*Significant effects are marked with bold font*.

**Table 6:** Results of pairwise comparisons on tone accuracy in Cues * Bands * Tones three-way repeated measures ANOVA by Backgrounds.

**No**.	**Background**	**Cue**	**Band**	**Tone order**	**Mean (SD)**					
					**T1**	**T2**	**T3**	**T4**	**T5**	**T6**
**1**	**QUI**	**ORI**	**B04**	T4(= T2 = T1 = T5)>(T2 = T1 = T5 =)T3>T6^a^						
**2**			**B08**		88% (13%)	88% (17%)	70% (17%)	98% (4%)	82% (21%)	42% (23%)
**3**			**B16**								
**4**		**TFS**	**B04**							
**5**			**B08**	T5 = T2(= T1 = T3)>(T1 = T3 =)T4>T6^a^	75% (20%)	86% (18%)	70% (21%)	59% (13%)	88% (19%)	18% (15%)
**6**			**B16**							
**7**		**TE500**	**B04**	T4>T2(= T3 = T5)>(T3 = T5 =)T1>T6^a^						
**8**			**B08**		27% (23%)	52% (26%)	30% (17%)	97% (5%)	25% (16%)	**5% (7%)**
**9**			**B16**							
**10**		**TE50**	**B04**	T4>T2(T1 = T3 = T5)>(T1 = T3 = T5 =)T6^a^						
**11**			**B08**		22% (25%)	36% (30%)	**15% (14%)**	86% (13%)	**12% (12%)**	**4% (7%)**
**12**			**B16**							
**13**	**SSN**	**ORI**	**B04**	T4>T1 = T3 = T5 = T2>T6^a^						
**14**			**B08**		79% (16%)	67% (22%)	77% (24%)	99% (4%)	73% (24%)	35% (25%)
**15**			**B16**							
**16**		**TFS**	**B04**	T1(= T3 = T5)> (T3 = T5 =)T4 = T2>T6^a^						
**17**			**B08**		77% (14%)	55% (23%)	69% (21%)	62% (15%)	60% (24%)	29% (21%)
**18**			**B16**							
**19**		**TE500**	**B04**	T4>T3 = T5(= T2 = T1)>(T2 = T1 =)T6^a^						
**20**			**B08**		19% (22%)	24% (19%)	24% (20%)	76% (15%)	21% (13%)	**8% (8%)**
**21**			**B16**							
**22**		**TE50**	**B04**	T4>T1 = T2 = T3 = T5 = T6^a^						
**23**			**B08**		19% (20%)	19% (16%)	17% (17%)	55% (21%)	**16% (12%)**	**6% (7%)**
**24**			**B16**							
**25**	**2MB**	**ORI**	**B04**	T4(= T1 = T2 = T5)>(T1 = T2 = T5) = T3>T6^b^	80% (23%)	80% (38%)	74% (24%)	98% (8%)	80% (26%)	35% (27%)
**26**			**B08**	T4(= T2 = T1)>(= T2 = T1) = T3>(= T3) = T5 = T6^b^	80% (23%)	83% (17%)	70% (30%)	98% (8%)	50% (38%)	46% (36%)
**27**			**B16**	T4(= T1)>(T1 =)T2(= T5 = T3)>(T5 = T3 =)T6^b^	78% (23%)	69% (21%)	46% (36%)	96% (11%)	59% (27%)	35% (29%)
**28**		**TFS**	**B04**	T5 = T4 = T2 = T1 = T3>T6^b^	72% (24%)	76% (28%)	65% (29%)	80% (23%)	81% (23%)	20% (26%)
**29**			**B08**	T1 = T4 = T2(= T5 = T3)>(T5 = T3 =)T6^b^	76% (30%)	65% (24%)	56% (30%)	70% (25%)	59% (31%)	30% (32%)
**30**			**B16**	T5 = T2(= T1 = T3 = T6)>(T1 = T3 = T6 =)T4, T5>T6^b^	48% (33%)	48% (26%)	39% (29%)	22% (23%)	61% (29%)	22% (32%)
**32**		**TE500**	**B04**	T4>T2 = T5 = T3 = T1 = T6^b^	**9% (19%)**	20% (28%)	**15% (21%)**	70% (25%)	19% (21%)	**9% (15%)**
**33**			**B08**	T4>T2 = T5 = T3 = T1 = T6^b^	**17% (26%)**	22% (26%)	**17% (24%)**	56% (28%)	20% (23%)	**9% (15%)**
**34**			**B16**	T4>T3(= T5 = T1 = T2)>(T5 = T1 = T2 =)T6^b^	20% (23%)	**17% (17%)**	31% (24%)	83% (24%)	26% (27%)	**4% (11%)**
**35**		**TE50**	**B04**	T4(= T3 = T1 = T5 = T2)>(T3 = T1 = T5 = T2 =)T6^b^	**17% (29%)**	**11% (16%)**	19% (23%)	33% (30%)	**15% (17%)**	**2% (8%)**
**36**			**B08**	T4>T5 = T2 = T1 = T3 = T6^b^	**15% (29%)**	20% (28%)	**11% (20%)**	61% (31%)	22% (20%)	**9% (19%)**
**37**			**B16**	T4>T5 = T2 = T3 = T1 = T6^b^	**11% (20%)**	19% (33%)	**11% (20%)**	72% (26%)	26% (29%)	**9% (15%)**

*Green cells mark T4 with the highest and T6 with the lowest accuracy*.

***Bold** numbers mark chance accuracy (17%) or below*.

a For QUI and SSN, tone orders with the Bands collapsed were reported because of the significant three-way Cues * Bands * Tones interactions and insignificant two-way Bands * Tones interactions (Table 3).

b For 2MB, tone orders for each Band were reported because of the significant three-way Cues * Bands * Tones interactions (Table 3), and the significant Bands * Tones interactions in each Cue in the two-way ANOVAs (Table 4).

**Table 7:** Results of pairwise comparisons in Cues * Bands * Tones three-way repeated measures ANOVA on tone accuracy by Backgrounds.

**No**.	**Background**	**Cue**	**Tone**	**Band order**	**Mean (SD)**		
					**B04**	**B08**	**B16**
**1**	**QUI**	**ORI**	**T1**	Band08 = Band16 = Band04^a^	76% (11%)	79% (10%)	79% (10%)
**2**			**T2**							
**3**			**T3**							
**4**			**T4**							
**5**			**T5**							
**6**			**T6**							
**7**		**TFS**	**T1**	Band08(= Band04)>(Band04 =)Band16^a^	66% (11%)	71% (10%)	62% (9%)
**8**			**T2**				
**9**			**T3**				
**10**			**T4**				
**11**			**T5**				
**12**			**T6**				
**13**		**TE500**	**T1**	Band04 = Band16 = Band08^a^	40% (12%)	38% (11%)	39% (8%)
**14**			**T2**				
**15**			**T3**				
**16**			**T4**				
**17**			**T5**				
**18**			**T6**				
**19**		**TE50**	**T1**	Band16(= Band08)>(Band08 =)Band04^a^	24% (10%)	29% (10%)	34% (9%)
**20**			**T2**				
**21**			**T3**				
**22**			**T4**				
**23**			**T5**				
**24**			**T6**				
**25**	**SSN**	**ORI**	**T1**	Band08 = Band04 = Band16^a^	72% (14%)	73% (13%)	69% (12%)
**26**			**T2**				
**27**			**T3**				
**28**			**T4**				
**29**			**T5**				
**30**			**T6**				
**31**		**TFS**	**T1**	Band04 = Band08>Band16^a^	66% (13%)	58% (11%)	51% (10%)
**32**			**T2**				
**33**			**T3**				
**34**			**T4**				
**35**			**T5**				
**36**			**T6**				
**37**		**TE500**	**T1**	Band16 = Band08 = Band04^a^	27% (10%)	29% (11%)	30% (5%)
**38**			**T2**				
**39**			**T3**				
**40**			**T4**				
**41**			**T5**				
**42**			**T6**				
**43**		**TE50**	**T1**	Band16 = Band08 = Band04^a^	20% (9%)	22% (8%)	24% (11%)
**44**			**T2**				
**45**			**T3**				
**46**			**T4**				
**47**			**T5**				
**48**			**T6**				
**49**	**2MB**	**ORI**	**T1**	Band04 = Band08 = Band16^b^	80%(23%)	80%(23%)	78%(23%)
**50**			**T2**	Band08(= Band04)>(Band04 =)Band16^b^	80%(38%)	83%(17%)	69%(21%)
**51**			**T3**	Band04 = Band08>Band16^b^	74%(24%)	70%(30%)	46%(36%)
**52**			**T4**	Band04 = Band08 = Band16^b^	98%(8%)	98%(8%)	96%(11%)
**53**			**T5**	Band04>Band16 = Band08^b^	80%(26%)	50%(38%)	59%(27%)
**54**			**T6**	Band08 = Band04 = Band16^b^	35%(27%)	46%(36%)	35%(29%)
**55**		**TFS**	**T1**	Band08(= Band04)>(Band04 =)Band16^b^	72%(24%)	76%(30%)	48%(33%)
**56**			**T2**	Band04(= Band08)>(Band08 =)Band16^b^	76%(28%)	65%(24%)	48%(26%)
**57**			**T3**	Band04 = Band08 = Band16^b^	65%(29%)	56%(30%)	39%(29%)
**58**			**T4**	Band04 = Band08>Band16^b^	80%(23%)	70%(25%)	22%(23%)
**59**			**T5**	Band04(= Band16)>(Band16 =)Band08^b^	81%(23%)	59%(31%)	61%(29%)
**60**			**T6**	Band08 = Band16 = Band04^b^	20%(26%)	30%(32%)	22%(32%)
**61**		**TE500**	**T1**	Band16 = Band08 = Band04^b^	**9%(19%)**	**17%(26%)**	20%(23%)
**62**			**T2**	Band08 = Band04 = Band16^b^	20%(28%)	22%(26%)	**17%(17%)**
**63**			**T3**	Band16 = Band08 = Band04^b^	**15%(21%)**	**17%(24%)**	31%(24%)
**64**			**T4**	Band16(= Band04)>(Band04 =)Band08^b^	70%(25%)	56%(28%)	83%(24%)
**65**			**T5**	Band16 = Band08 = Band04^b^	19%(21%)	20%(23%)	26%(27%)
**66**			**T6**	Band04 = Band08 = Band16^b^	**9%(15%)**	**9%(15%)**	**4%(11%)**
**67**		**TE50**	**T1**	Band04 = Band08 = Band16^b^	**17%(29%)**	**15%(29%)**	**11%(20%)**
**68**			**T2**	Band08 = Band16 = Band04^b^	**11%(16%)**	20%(28%)	19%(33%)
**69**			**T3**	Band04 = Band16 = Band08^b^	19%(23%)	**11%(20%)**	**11%(20%)**
**70**			**T4**	Band16 = Band08>Band04^b^	33%(30%)	61%(31%)	72%(26%)
**71**			**T5**	Band16 = Band08 = Band04^b^	**15%(17%)**	22%(20%)	26%(29%)
**72**			**T6**	Band08 = Band16 = Band04^b^	**2%(8%)**	**9%(19%)**	**9%(15%)**

Beige cells mark Band04 = Band08 = Band16, in any order. Peach cells

mark lowest tone identification accuracies in B16.

Blue cells mark higher accuracies with more number of bands (i.e., in the order of Band16, Band08, Band04).

**Bold** numbers mark chance accuracy (17%) or below.

a For QUI and SSN, orders of Bands collapsing the Tones were reported because of the non-significant three-way Cues * Bands * Tones interactions and the non-significant two-way Bands * Tones interactions (Table 3).

b For 2MB, Band orders for each Tone were reported because of the significant Cues * Bands * Tones three-way interactions (Table 3), and the significant Bands *Tones interactions in each Cue in two-way ANOVAs (Table 4).

**Table 8:** Results of pairwise comparisons in Tones * Bands * Cue three-way repeated measures ANOVA on tone accuracy.

**No**.	**Background**	**Tone**	**Band**	**Cue order**	**Mean (SD)**			
					**ORI**	**TFS**	**TE500**	**TE50**
**1**	**QUI**	**T1**	**B04**	ORI = TFS>TE500>TE50^a^	83% (26%)	72% (29%)	30% (32%)	**13% (31%)**
**2**			**B08**	ORI = TFS>TE500 = TE50^a^	87% (17%)	72% (35%)	31% (35%)	22% (30%)
**3**			**B16**	ORI = TFS>TE50 = TE500^a^	93% (14%)	81% (21%)	19% (26%)	30% (34%)
**4**		**T2**	**B04**	ORI = TFS>TE500>TE50^a^	83% (26%)	81% (29%)	50% (33%)	24% (28%)
**5**			**B08**	TFS = ORI>TE500 = TE50^a^	91% (15%)	93% (14%)	48% (33%)	35% (42%)
**6**			**B16**	ORI = TFS>TE500 = TE50^a^	91% (19%)	83% (24%)	57% (34%)	48% (37%)
**7**		**T3**	**B04**	ORI = TFS>TE500 = TE50^a^	69% (29%)	63% (34%)	30% (32%)	**17% (26%)**
**8**			**B08**	TFS = ORI>TE500 = TE50^a^	70% (30%)	83% (29%)	22% (20%)	**9% (19%)**
**9**			**B16**	ORI = TFS>TE500 = TE50^a^	72% (33%)	65% (27%)	37% (23%)	19% (23%)
**10**		**T4**	**B04**	ORI = TE500(= TE50)>(TE50 =)TFS^a^	100% (0%)	69% (18%)	98% (8%)	83% (26%)
**11**			**B08**	ORI = TE500 = TE50>TFS^a^	98% (8%)	61% (21%)	98% (8%)	89% (16%)
**12**			**B16**	ORI = TE500 = TE50>TFS ^a^	96% (11%)	48% (21%)	94% (13%)	87% (20%)
**13**		**T5**	**B04**	TFS = ORI>TE500 = TE50^a^	80% (28%)	89% (26%)	28% (29%)	**9% (15%)**
**14**			**B08**	TFS = ORI>TE500 = TE50^a^	83% (21%)	91% (19%)	24% (28%)	**13% (20%)**
**15**			**B16**	TFS = ORI>TE500 = TE50^a^	83% (26%)	85% (26%)	22% (23%)	**15% (21%)**
**16**		**T6**	**B04**	ORI(= TFS)>(TFS =)(= TE500)>(TE500 =)TE50^a^	39% (33%)	22% (28%)	**7% (14%)**	**0% (0%)**
**17**			**B08**	ORI(= TFS)>(TFS =)(= TE50)>(TE50 =)TE500^a^	46% (28%)	24% (25%)	**4% (11%)**	**7% (18%)**
**18**			**B16**	ORI>TFS = TE50 = TE500^a^	41% (33%)	**7% (14%)**	**4% (11%)**	**4% (11%)**
**19**	**SSN**	**T1**	**B04**	TFS = ORI>TE500 = TE50^a^	80% (20%)	87% (23%)	**17% (29%)**	**9% (19%)**
**20**			**B08**	ORI = TFS>TE500 = TE50^a^	81% (23%)	74% (18%)	22% (30%)	**13% (23%)**
**21**			**B16**	ORI = TFS>TE50 = TE500^a^	76% (25%)	70% (23%)	19% (26%)	35% (37%)
**22**		**T2**	**B04**	ORI = TFS>TE500 = TE50^a^	69% (29%)	56% (36%)	22% (23%)	**13% (17%)**
**23**			**B08**	ORI = TFS>TE50 = TE500^a^	67% (23%)	61% (26%)	22% (26%)	22% (20%)
**24**			**B16**	ORI(= TFS)>(TFS =)(= TE500)>(TE500 =)TE50^a^	67% (34%)	48% (31%)	28% (31%)	20% (23%)
**25**		**T3**	**B04**	ORI = TFS>TE50 = TE500^a^	72% (33%)	69% (31%)	22% (20%)	22% (32%)
**26**			**B08**	ORI = TFS>TE500 = TE50^a^	78% (30%)	70% (32%)	28% (26%)	20% (26%)
**27**			**B16**	ORI = TFS>TE500 = TE50^a^	80% (33%)	67% (23%)	22% (26%)	**7% (18%)**
**28**		**T4**	**B04**	ORI(= TFS)>(TFS =)(= TE500)>(TE500 =)TE50^a^	98% (8%)	83% (24%)	74% (22%)	56% (26%)
**29**			**B08**	ORI>TE500 = TFS = TE50^a^	98% (8%)	59% (24%)	74% (22%)	54% (28%)
**30**			**B16**	ORI>TE500(= TE50)>(TE50 =)TFS^a^	100% (0%)	43% (22%)	80% (28%)	56% (32%)
**31**		**T5**	**B04**	TFS = ORI>TE500 = TE50^a^	74% (29%)	74% (29%)	**17% (24%)**	**15% (23%)**
**32**			**B08**	ORI = TFS>TE500 = TE50^a^	80% (31%)	52% (37%)	20% (26%)	**13% (17%)**
**33**			**B16**	ORI = TFS>TE500 = TE50^a^	65% (35%)	54% (31%)	26% (22%)	20% (23%)
**34**		**T6**	**B04**	ORI(= TFS)>(TFS =)(= TE500)>(TE500 =)TE50^a^	39% (37%)	30% (25%)	**9% (15%)**	**6% (13%)**
**35**			**B08**	ORI = TFS>TE50 = TE500^a^	35% (35%)	33% (26%)	**7% (14%)**	**7% (14%)**
**36**			**B16**	ORI(= TFS)>(TFS =)TE500 = TE50^a^	30% (28%)	24% (28%)	**7% (18%)**	**4% (11%)**
**37**	**2MB**	**T1**	**B04**	ORI = TFS>TE50 = TE500^b^	80% (23%)	72% (24%)	9% (19%)	**17% (29%)**
**38**			**B08**	ORI = TFS>TE500 = TE50^b^	80% (23%)	76% (30%)	**17% (26%)**	**15% (29%)**
**39**			**B16**	ORI>TFS>TE500 = TE50^b^	78% (23%)	48% (33%)	20% (23%)	**11% (20%)**
**40**		**T2**	**B04**	ORI = TFS>TE500 = TE50^b^	80% (38%)	76% (28%)	20% (28%)	**11% (16%)**
**41**			**B08**	ORI>TFS>TE500 = TE50^b^	83% (17%)	65% (24%)	22% (26%)	20% (28%)
**42**			**B16**	ORI(= TFS)>(TFS =)TE50 = TE500^b^	69% (21%)	48% (26%)	**17% (17%)**	19% (33%)
**43**		**T3**	**B04**	ORI = TFS>TE50 = TE500^b^	74% (24%)	65% (29%)	**15% (21%)**	19% (23%)
**44**			**B08**	ORI = TFS>TE500 = TE50^b^	70% (30%)	56% (30%)	**17% (24%)**	**11% (20%)**
**45**			**B16**	ORI = TFS = TE500>TE50^b^	46% (36%)	39% (29%)	31% (24%)	**11% (20%)**
**46**		**T4**	**B04**	ORI>TFS = TE500>TE50^b^	98% (8%)	80% (23%)	70% (25%)	33% (30%)
**47**			**B08**	ORI>TFS = TE50 = TE500^b^	98% (8%)	70% (25%)	56% (28%)	61% (31%)
**48**			**B16**	ORI(= TE500)>(TE500 =)TE50>TFS^b^	96% (11%)	22% (23%)	83% (24%)	72% (26%)
**49**		**T5**	**B04**	TFS = ORI>TE500 = TE50^b^	80% (26%)	81% (23%)	19% (21%)	**15% (17%)**
**50**			**B08**	TFS(= ORI = TE50)>(ORI = TE50 =)TE500^b^	50% (38%)	59% (31%)	20% (23%)	22% (20%)
**51**			**B16**	TFS = ORI>TE50 = TE500^b^	59% (27%)	61% (29%)	26% (27%)	26% (29%)
**52**		**T6**	**B04**		39% (24%)	24% (23%)	**7% (9%)**	**7% (9%)**
**53**			**B08**	ORI >TFS>TE500 = TE50^c^				
**54**			**B16**					

Blue cells mark “ORI = TFS>TE500 = TE50,” with ORI and TFS, and TE500 and TE50 in any order.

Pink cells mark ORI significantly better than TFS, TE500 and TE50.

Beige cells mark TFS as the worst.

Peach cells mark TE50 as the worst.

Unformatted cells mark other orders.

**Bold** cells mark chance accuracy (17%) or below.

a For QUI and SSN, orders of Cues in each band were reported because of the insignificant three-way Cues * Bands * Tones interactions, and the significant two-way Cues * Bands and Cues * Tones interactions (Table 3).

b For T1, T2, T3, T4, and T5 in 2MB, Cue orders for each Tone were reported because of the significant Cues * Bands * Tones three-way interactions (Table 3), and the significant Bands * Tones interactions in the two-way ANOVA of Bands * Cues separated by Tones (Table 5).

c For T6 in 2MB, Cue orders with the Tones collapsed were reported because of the significant Cues * Bands * Tones three-way interactions (Table 3), and the insignificant Bands * Tones interactions in the two-way ANOVA of Bands * Cues separated by Tones (Table 5).

**Table 9:** Results of Tone * Bands * Backgrounds three-way repeated measures ANOVA on tone accuracy by Cues.

**Cue**		**df1**	**df2**	***F***	***p***	**Partial η^2^**
ORI	**Backgrounds**	2.00	34.00	19.14	**<0.001**	0.53
	Bands	2.00	34.00	1.96	0.157	0.10
	**Tones**	3.14	53.41	27.16	**<0.001**	0.62
	**Backgrounds** * **Bands**	4.00	68.00	2.69	**0.038**	0.14
	**Backgrounds** * **Tones**	10.00	170.00	4.66	**<0.001**	0.22
	Bands * Tones	10.00	170.00	0.88	0.555	0.05
	Backgrounds * Bands * Tones	8.19	139.15	1.59	0.130	0.09
TFS	**Backgrounds**	2.00	34.00	22.57	**<0.001**	0.57
	**Bands**	2.00	34.00	34.85	**<0.001**	0.67
	**Tones**	3.47	58.96	28.22	**<0.001**	0.62
	**Backgrounds** * **Bands**	4.00	68.00	6.64	**<0.001**	0.28
	**Backgrounds** * **Tones**	10.00	170.00	8.49	**<0.001**	0.33
	**Bands** * **Tones**	10.00	170.00	4.62	**<0.001**	0.21
	Backgrounds * Bands * Tones	8.17	138.90	1.83	0.074	0.10
TE500	**Backgrounds**	2.00	34.00	48.75	**<0.001**	0.74
	Bands	2.00	34.00	1.36	0.271	0.07
	**Tones**	2.83	48.06	61.04	**<0.001**	0.78
	Backgrounds * Bands	4.00	68.00	1.49	0.214	0.08
	**Backgrounds** * **Tones**	10.00	170.00	6.28	**<0.001**	0.27
	Bands * Tones	5.58	94.78	1.18	0.324	0.06
	Backgrounds * Bands * Tones	9.03	153.48	1.24	0.272	0.07
TE50	**Backgrounds**	2.00	34.00	8.83	**<0.001**	0.34
	**Bands**	2.00	34.00	9.76	**<0.001**	0.36
	**Tones**	2.27	38.62	40.99	**<0.001**	0.71
	Backgrounds * Bands	4.00	68.00	0.96	0.436	0.05
	**Backgrounds** * **Tones**	10.00	170.00	7.70	**<0.001**	0.31
	**Bands** * **Tones**	10.00	170.00	2.21	**0.019**	0.12
	**Backgrounds** * **Bands** * **Tones**	9.20	156.32	2.20	**0.024**	0.11

*Significant effects are marked with bold font*.

**Table 10:** Results of Backgrounds * Bands two-way repeated measures ANOVA on tone accuracy by Tones when Cue = TE50.

**Cue**	**Tone**		**df1**	**df2**	**F**	***p***	**Partial η^2^**
**TE50**	**T1**	Backgrounds	2.00	34.00	1.43	0.252	0.08
		**Bands**	2.00	34.00	4.01	**0.027**	0.19
		**Backgrounds** * **Bands**	4.00	68.00	3.55	**0.011**	0.17
	**T2**	**Backgrounds**	2.00	34.00	7.12	**0.003**	0.30
		**Bands**	2.00	34.00	7.94	**0.001**	0.32
		Backgrounds * Bands	4.00	68.00	1.11	0.358	0.06
	**T3**	Backgrounds	2.00	34.00	0.23	0.793	0.01
		Bands	2.00	34.00	1.08	0.352	0.06
		Backgrounds * Bands	4.00	68.00	1.35	0.260	0.07
	**T4**	**Backgrounds**	2.00	34.00	30.01	**<0.001**	0.64
		**Bands**	2.00	34.00	5.15	**0.011**	0.23
		**Backgrounds** * **Bands**	4.00	68.00	3.53	**0.011**	0.17
	**T5**	Backgrounds	2.00	34.00	1.92	0.162	0.10
		Bands	2.00	34.00	1.56	0.225	0.08
		Backgrounds * Bands	4.00	68.00	0.33	0.857	0.02
	**T6**	Backgrounds	2.00	34.00	0.78	0.466	0.04
		Bands	2.00	34.00	1.95	0.158	0.10
		Backgrounds * Bands	4.00	68.00	0.65	0.626	0.04

*Significant effects are marked with bold font*.

**Table 11:** Results of pairwise comparisons in Tones * Bands * Backgrounds three-way repeated measures ANOVA on tone accuracy.

**No**	**Cue**	**Tone**	**Band**	**Background Order**	**Mean (SD)**		
					**QUI**	**SSN**	**2MB**
**1**	**ORI**	**T1**	**B04**	QUI = 2MB = SSN^a^	83% (26%)	80% (20%)	80% (23%)
**2**			**B08**	QUI = SSN = 2MB^a^	87% (17%)	81% (23%)	80% (23%)
**3**			**B16**	QUI(= 2MB)>(2MB =)SSN^a^	93% (14%)	76% (25%)	78% (23%)
**4**		**T2**	**B04**	QUI = 2MB = SSN^a^	83% (26%)	69% (29%)	80% (38%)
**5**			**B08**	QUI = 2MB>SSN^a^	91% (15%)	67% (23%)	83% (17%)
**6**			**B16**	QUI>2MB = SSN^a^	91% (19%)	67% (34%)	69% (21%)
**7**		**T3**	**B04**	2MB = SSN = QUI^a^	69% (29%)	72% (33%)	74% (24%)
**8**			**B08**	SSN = 2MB = QUI^a^	70% (30%)	78% (30%)	70% (30%)
**9**			**B16**	SSN = QUI = 2MB^a^	72% (33%)	80% (33%)	46% (36%)
**10**		**T4**	**B04**	QUI = 2MB = SSN^a^	100% (0%)	98% (8%)	98% (8%)
**11**			**B08**	2MB = QUI = SSN^a^	98% (8%)	98% (8%)	98% (8%)
**12**			**B16**	SSN = 2MB = QUI^a^	96% (11%)	100% (0%)	96% (11%)
**13**		**T5**	**B04**	2MB = QUI = SSN^a^	80% (28%)	74% (29%)	80% (26%)
**14**			**B08**	QUI = SSN>2MB^a^	83% (21%)	80% (31%)	50% (38%)
**15**			**B16**	QUI(= SSN)>(SSN =)2MB^a^	83% (26%)	65% (35%)	59% (27%)
**16**		**T6**	**B04**	QUI = SSN = 2MB^a^	39% (33%)	39% (37%)	35% (27%)
**17**			**B08**	QUI = 2MB = SSN^a^	46% (28%)	35% (35%)	46% (36%)
**18**			**B16**	QUI = 2MB = SSN^a^	41% (33%)	30% (28%)	35% (29%)
**19**	**TFS**	**T1**	**B04**	SSN(= 2MB)>(2MB =)QUI^a^	72% (29%)	87% (23%)	72% (24%)
**20**			**B08**	2MB = SSN = QUI^a^	72% (35%)	74% (18%)	76% (30%)
**21**			**B16**	QUI = SSN>2MB^a^	81% (21%)	70% (23%)	48% (33%)
**22**		**T2**	**B04**	QUI = 2MB>SSN^a^	81% (29%)	56% (36%)	76% (28%)
**23**			**B08**	QUI>2MB = SSN^a^	93% (14%)	61% (26%)	65% (24%)
**24**			**B16**	QUI>2MB = SSN^a^	83% (24%)	48% (31%)	48% (26%)
**25**		**T3**	**B04**	SSN = 2MB = QUI^a^	63% (34%)	69% (31%)	65% (29%)
**26**			**B08**	QUI(= SSN)>(SSN =)2MB^a^	83% (29%)	70% (32%)	56% (30%)
**27**			**B16**	SSN = QUI>2MB^a^	65% (27%)	67% (23%)	39% (29%)
**28**		**T4**	**B04**	SSN = 2MB = QUI^a^	69% (18%)	83% (24%)	80% (23%)
**29**			**B08**	2MB = QUI = SSN^a^	61% (21%)	59% (24%)	70% (25%)
**30**			**B16**	QUI = SSN>2MB^a^	48% (21%)	43% (22%)	22% (23%)
**31**		**T5**	**B04**	QUI = 2MB = SSN^a^	89% (26%)	74% (29%)	81% (23%)
**32**			**B08**	QUI>2MB = SSN^a^	91% (19%)	52% (37%)	59% (31%)
**33**			**B16**	QUI(= 2MB)>(2MB =)SSN^a^	85% (26%)	54% (31%)	61% (29%)
**34**		**T6**	**B04**	SSN = QUI = 2MB^a^	22% (28%)	30% (25%)	20% (26%)
**35**			**B08**	SSN = 2MB = QUI^a^	24% (25%)	33% (26%)	30% (32%)
**36**			**B16**	SSN = 2MB = QUI^a^	**7% (14%)**	24% (28%)	22% (32%)
**37**	**TE500**	**T1**	**B04**	QUI(= SSN)>(SSN =)2MB^b^	27% (23%)	19% (22%)	**15% (18%)**
**38**			**B08**				
**39**			**B16**				
**40**		**T2**	**B04**	QUI>SSN = 2MB^b^	52% (26%)	24% (19%)	20% (18%)
**41**			**B08**				
**42**			**B16**				
**43**		**T3**	**B04**	QUI = SSN = 2MB^b^	30% (17%)	24% (20%)	21% (17%)
**44**			**B08**				
**45**			**B16**				
**46**		**T4**	**B04**	QUI>SSN = 2MB^b^	97% (5%)	76% (15%)	70% (15%)
**47**			**B08**				
**48**			**B16**				
**49**		**T5**	**B04**	QUI = 2MB = SSN^b^	25% (16%)	21% (13%)	22% (13%)
**50**			**B08**				
**51**			**B16**				
**52**		**T6**	**B04**	SSN = 2MB = QUI^b^	**5% (7%)**	**8% (8%)**	**7% (9%)**
**53**			**B08**				
**54**			**B16**				
**55**	**TE50**	**T1**	**B04**	2MB = SSN = QUI^c^	**13% (31%)**	**9% (19%)**	**17% (29%)**
**56**			**B08**	QUI = 2MB = SSN^c^	22% (30%)	**13% (23%)**	**15% (29%)**
**57**			**B16**	SSN(= QUI)>(QUI =)2MB^c^	30% (34%)	35% (37%)	**11% (20%)**
**58**		**T2**	**B04**	QUI>SSN = 2MB^d^	36% (30%)	**19% (16%)**	**17% (21%)**
**59**			**B08**				
**60**			**B16**				
**61**		**T3**	**B04**	SSN = QUI = 2MB^d^	**15% (14%)**	**17% (17%)**	**14% (12%)**
**62**			**B08**				
**63**			**B16**				
**64**		**T4**	**B04**	QUI>SSN>2MB^c^	83% (26%)	56% (26%)	33% (30%)
**65**			**B08**	QUI>2MB = SSN^c^	89% (16%)	54% (28%)	61% (31%)
**66**			**B16**	QUI(= 2MB)>(2MB =)SSN^c^	87% (20%)	56% (32%)	72% (26%)
**67**		**T5**	**B04**	2MB = SSN = QUI^d^	**12% (12%)**	**16% (12%)**	**21% (15%)**
**68**			**B08**				
**69**			**B16**				
**70**		**T6**	**B04**				
**71**			**B08**	2MB = SSN = QUI^d^	**4% (7%)**	**6% (7%)**	**7% (9%)**
**72**			**B16**				

*Green cells mark SSN = 2MB = QUI, in any order*.

Beige cells mark accuracy in QUI higher than both SSN and 2MB.

Blue cells mark higher accuracy in QUI than either SSN or 2MB.

**Bold** cells mark chance accuracy (17%) or below.

a For ORI and TFS, orders of Background in each band were reported because of the significant three-way Backgrounds * Bands * Tones interactions, and the significant two-way Backgrounds * Bands and Backgrounds * Tones interactions (Table 9).

b For TE500, orders of Background with the Bands collapsed were reported because of the significant three-way Backgrounds * Bands * Tones interactions, the significant two-way Backgrounds * Tones interactions and the non-significant Backgrounds * Bands interactions (Table 9).

c For T1 and T4 in TE50, orders of Background in each band were reported because of the significant three-way Backgrounds * Bands * Tones interactions (Table 9), the significant two-way Backgrounds * Bands interactions in the two-way Backgrounds * Bands ANOVA separated by Tones in Table 10.

d For T2, T3, T5, and T6 in TE50, orders of Background with the Bands collapsed were reported because of the significant three-way Backgrounds * Bands * Tones interactions (Table 9), and the non-significant two-way Backgrounds * Bands interactions in the two-way Backgrounds * Bands ANOVA separated by Tones (Table 10).

For all analyses, the significance level was set at 0.05 and corrected with Bonferroni adjustments for multiple comparisons. To indicate the order of accuracy, significant differences were marked by “>” or “ < .” Non-significant differences were marked by “ = .” When a condition was not significantly different from two other conditions that were significantly different from each other, it was put in parenthesis and repeated on both side of the “>” or “ < ” sign. Example, “T2 (= T3) > (T3 =) T6” means T2 was significantly larger than T6, while T3 was not significantly different from either T2 or T6. Means and standard deviations (SDs) of accuracies at or below chance level were marked in bold form in the tables.

### Tone identification accuracies in the original stimuli in quiet

Before assessing how effective the temporal cues were for Cantonese tone identification, we first examined how well Cantonese tones were identified in naturally produced original stimuli in quiet and noise conditions.

Figure 1 shows that in quiet, all tones, except T6 (LL), were identified with high accuracy in the original signal. There was little effect of number of bands. Table 6 (rows 1-3) shows that when listeners listened to the naturally produced stimuli in quiet, they identified T1 (HL), T2 (HR), T4 (LF), and T5 (LR) with 82–98% accuracy and T3 (ML) and T6 (LL) with 70 and 42% accuracy, respectively. T4 (LF) was the easiest and T6 (LL) was the most difficult to identify. The non-significant interactions between tone accuracy and Bands in quiet (Table 3) indicated that number of bands did not affect tone identification in the original stimuli in quiet.

Together the findings showed that listeners were able to identify most of the tones in natural stimuli with high accuracy in quiet. Number of frequency bands did not affect Cantonese tone perception in the original signal in quiet.

### Tone identification accuracies in original stimuli in noise

Figure 1 shows that tone accuracies in the original signal in noise appeared to be comparable to those in quiet. Table 6 (rows 13–15 and 25–27) shows that in noise, most tones presented in the original signal continued to be identified with high accuracy. T4 (LF) and T6 (LL) in the original signal in noise were identified with the highest and lowest accuracy, respectively, following the pattern in the original signal in quiet.

To determine if number of frequency bands affected tone identification in the original signal in noise, pairwise comparisons were conducted on tone accuracy in different number of Bands. Table 7 (rows 25–30) confirmed no effect of number of bands on tone identification in the original signal in SSN. Table 7 (rows 49–54) shows interactions of Tones and Bands on tone identification in the original signal in 2MB. However, in half of the cases, tone accuracies were comparable in all number of frequency Bands (cells highlighted in beige color). In other cases in which Bands had significant effects on tone accuracy in the original signal, tone accuracy did not appear to change consistently with an increase or decrease in the number of Bands.

To determine if tone identification accuracies in the original signal were significantly affected by noise, a three-way repeated measures ANOVA (Tones × Bands × Backgrounds) was conducted for each of the four acoustic cues (Table 9). Results of pairwise comparisons presented in Table 11 (rows 1–18) showed that in most cases (cells highlighted in green), no significant difference was found in tone identification accuracies in the three backgrounds when the tones were presented in the original signal. Only T1 (HL) in B16, and T2 (HR) and T5 (LR) in B08 and B16 had lower accuracy in one or both types of noise than in quiet (Table 11). The findings indicated no strong effect of noise, or noise type on Cantonese tone perception in the original signal.

Together, the findings indicated that when listening to the original signal in noise, there was little effect of noise, noise type or number of bands on tone identification accuracy. Tone identification in the original signal in noise was mostly comparable to that in quiet, except that the two rising tones in B08 and B16 were perceived with significantly lower accuracy in one type of noise than in quiet.

### Tone identification accuracies in the three temporal cues in quiet

Figure 1 shows that among the three temporal cues, TFS was the best cue for tone identification in quiet, as indicated by much higher accuracies in TFS than in TE500 or TE50 in quiet, except for T4 (LF). TE500 and TE50 appeared to be weak cues for tone identification in quiet because identification accuracies for most of the tones, except for T2 (HR) and T4 (LF), were around chance level. T4 (LF) was the only tone that was identified with higher accuracy in TE500 and TE50 than in TFS when presented in quiet. Table 6 (rows 4–12) shows tone identification accuracies in the three temporal cues in quiet. T6 (LL) was the most difficult to identify (mean accuracies = 4–18%) in all three temporal cues, while T4 (LF) was the easiest to identify in TE500 (mean accuracy = 97%) and TE50 (mean accuracy = 86%), but the second most difficult tones to identify in TFS (mean accuracy = 59%). Results of pairwise comparisons on tone accuracy in different acoustic cues (Table 8 rows 1–18) confirmed that in the quiet condition, tone identification in TFS was comparable to that of the original stimuli, and significantly better than in TE500 or TE50, in all Band numbers, except for the identification of T4 (LF) in all bands (rows 10–12) and T6 in B16 (row 18). TE500 and TE50 were as effective as the original cue for T4 (LF) identification (Table 8, rows 10–12), but were weak for identifying the other tones, indicated by the low mean accuracies and the significantly lower accuracies than in TFS and the original stimuli. TE50 appeared to be the worst cue for tone identification in quiet given that identification accuracies for most of the tones in TE50 either had the lowest mean accuracies (Table 8, rows 1–18) or were significantly lower than those in TE500 (Table 8, rows 1 and 4).

Figure 1 shows small effects of frequency Bands on the temporal cues in quiet. Effects of Bands appeared to occur in T4 (LF) in TFS and T2 (HR) in TE50. Table 7 (rows 7–12 and rows 19–24) confirms the effects of Bands on TFS and TE50 in quiet. Tone identification accuracies in TFS were lower in B16 than in B08 in quiet (Table 7 rows 7–12). When presented in TE50 in quiet, tone identification accuracies were lower in B04 than in B16 (Table 7, rows 19–24). Table 8 (rows 10–12) indicates that when the tones were presented in TFS in 4 or 8 bands in quiet, even T4 (LF), the most difficult tone to identify in TFS and the only tone that was perceived with higher accuracies in TE500 and TE50 than in TFS, reached over 60% accuracy. For TE50, though tones presented in B16 in quiet were identified more correctly than tones presented in B04, even when the tones were presented with the highest number of bands, tone accuracies in TE50 in quiet were still lower than 50% (Table 8, last column, rows 1–18), except for T4 (LF).

Taken together, among the three temporal cues, TFS was the most effective cue for tone identification in quiet, particularly in fewer frequency bands. Cantonese tone identification in TFS in quiet was as effective as in the original signal, except for T4 (LF). When the tones were presented in TFS in fewer bands in quiet, identification accuracy of T4 (LF) reached over 60%. TE500 and TE50 were not effective cues for tone identification in quiet, except for T4 (LF). TE50 was the weakest temporal cue for Cantonese tone identification in quiet.

### Tone identification accuracies in the three temporal cues in noise

Figure 1 shows that tone identification accuracies of the three temporal cues in the two types of noise (2nd and 3rd columns) generally followed the patterns in quiet (1st column). Table 6 confirmed that orders of tone accuracy in different temporal cues in noise (rows 16–24 and 28–37) followed similar orders of accuracy as in quiet (Table 6, rows 4–12). In noise, T6 (LL) remained the most difficult tone to identify in all the three temporal cues, as in quiet. T4 (LF) was the easiest tone to perceive in TE500 and TE50 in noise, but was difficult to identify in TFS, except in B04 and B08 in 2MB. Table 8 (rows 19–54) shows that, among the three temporal cues, TFS remained the best cue for tone identification in noise, as indicated by the comparable accuracies between the original signal and TFS, and significantly higher tone accuracies in TFS than in TE500 or TE50 in most conditions (Table 8, rows 19–54, blue cells). In most of the other cases, accuracies in TFS was lower than in the original signal but were still higher than or equal to those in TE500 and/or TE50, except for T4 (LF) in B16 in both 2MB and SSN (Table 8 beige cells).

Table 7 shows significant effects of Bands on tone identification in TFS in noise. In SSN, tones in TFS were perceived with lower accuracies in B16 (Table 7, rows 31–36, peach color cells). In 2MB, tone identification accuracies in TFS also appeared to be lower in B16, as indicated by significantly poorer tone identification accuracies for T4 (LF), T1 (HL), and T2 (HR) in B16 than in B04 and/or B08 (Table 7, rows 55–60, peach color cells) and the lowest mean accuracy in B16 than the other two Bands for T3 (ML) (row 57). When the tones were presented in TFS in 4 bands, T4 (LF) reached mean accuracies of 83 and 80% in SSN and 2MB, respectively (Table 8, rows 28 and 46). No strong effect of Bands was found for TE500 and TE50 in noise (Table 7, rows 37–48 and 61–72).

Types of noise did not have a strong effect on tone accuracies presented in the three temporal cues. Table 11 (rows 19–72) shows that tone accuracies in 2MB and SSN were mostly comparable (cells in green and in beige). When tone accuracies were different in SSN and 2MB (cells in blue), no consistent order of difference was found with different tones and different bands.

Taken together, among the three temporal cues, TFS was the most effective cue for Cantonese tone identification in noise. Though T4 (LF) in noise was more difficult to identify in TFS, high identification accuracy of over 80% was achieved when T4 (LF) was presented in TFS in 4 frequency bands. TE500 and TE50 were not effective cues for Cantonese tone identification in noise, except for T4 (LF). Noise type did not have consistent effects on tone identification in the three temporal cues in noise.

### Identification of tone heights and tone shapes in the original cues in quiet and in noise

Error pattern analyses were carried out to examine the perception of tone height and tone shapes in different acoustic cues. Table 12 presents the confusion matrix of the three contour tones [i.e., T2 (HR), T5 (LR), T4 (LF)]. Table 13 presents the confusion matrix of the three level tones [i.e., T1 (HL), T3 (ML), T6 (LL)]. Target responses were highlighted with bold fonts and thick borders. Green cells mark the modal and correct responses. Peach cells mark the most frequent but incorrect responses. Beige cells mark non-modal responses that were higher than chance level (17%).

**Table 12:**
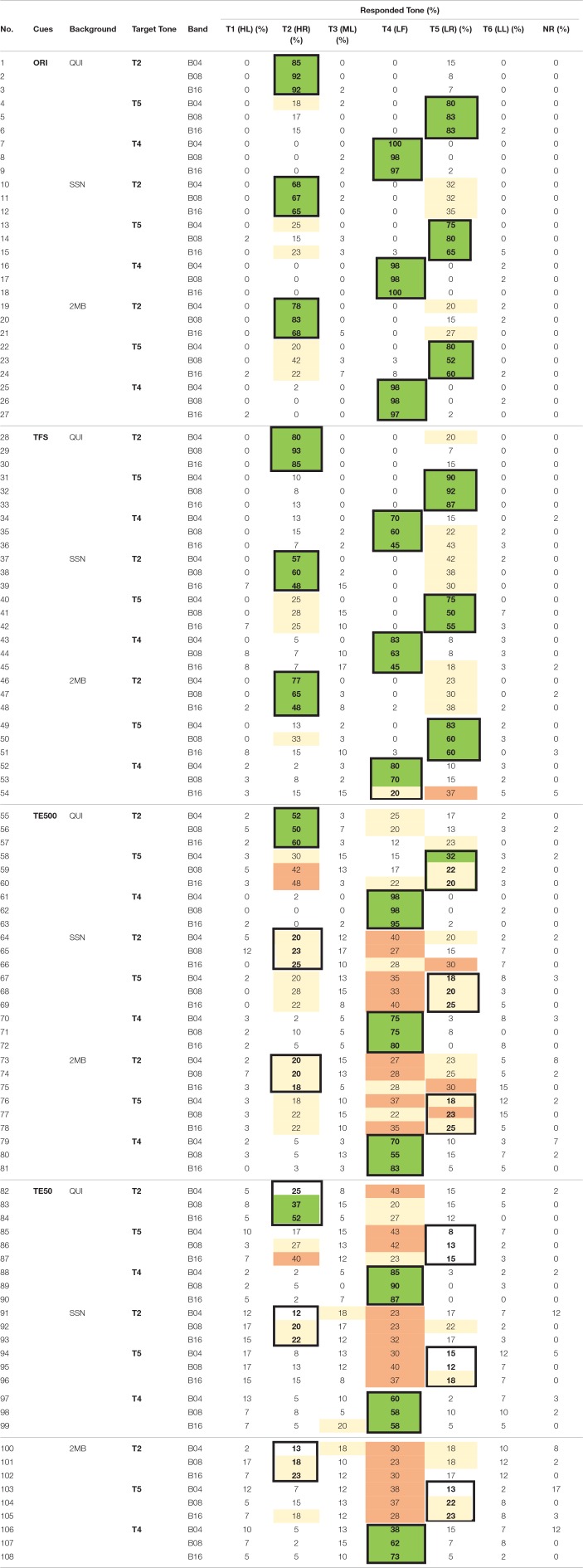
**Confusion matrix of the contour tones—T2 (HR), T5 (LR), T4 (LF)**.

Groups of cells with thick borders mark the target responses.

Green cells mark modal and correct responses.

Peach cells mark modal but incorrect responses.

Beige cells mark other responses above chance level (17%).

**Bold** cells mark correct responses.

**Table 13:**
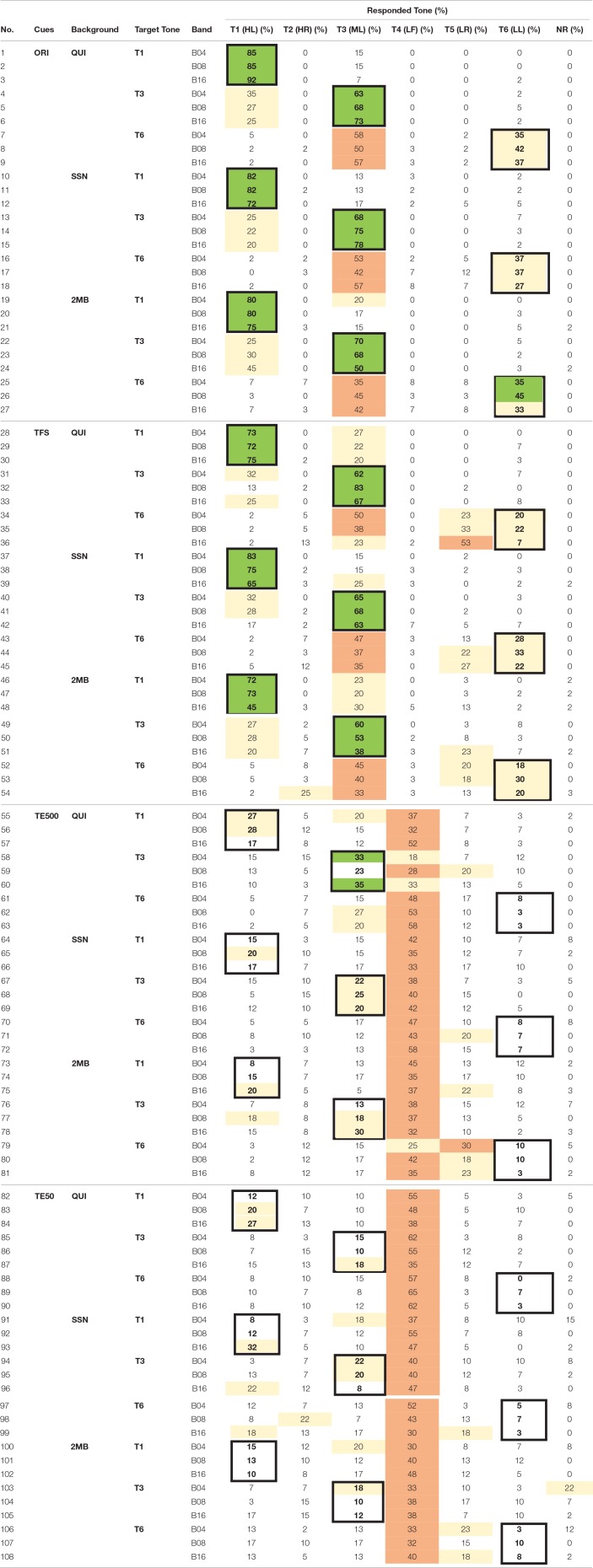
**Confusion matrix of the three level tones–T1 (HL), T3 (ML), T6 (LL)**.

Groups of cells with thick borders mark the target responses.

Green cells mark modal and correct responses.

Peach cells mark modal but incorrect responses.

Beige cells mark other responses above chance level (17%).

**Bold** cells mark correct responses.

Table 12 (rows 1–9) shows that when the tones were presented in the original signal in quiet, listeners were able to identify the shapes of the contour tones, as indicated by ceiling identification accuracy and little confusion between T2 (HR) and T5 (LR). Table 13 (rows 1–9) shows that listeners had more difficulty identifying tone heights even when tones were presented in quiet. Many T3 (ML) productions were mis-identified as T1 (HL) and most of T6 (LL) productions were mis-heard as T3 (ML).

When the tones in the original stimuli were presented in noise, there were more errors in the identification of the rising slopes. Tables 12 (rows 10–15, 19–24) shows that when the tones were presented in the original signal in noise, there were more bidirectional T2 (HR)–T5 (LR) confusions in SSN (rows 10–15) and 2MB (rows 19–24) than in QUI (rows 1–6). Table 13 (rows 10–18, 19–27) shows that the identification of T1 (HL), T3 (ML), and T6 (LL) in the original signal in noise followed similar error patterns as in quiet (rows 1–9), indicating relatively small effect of noise on the identification of tone heights in the three level tones.

Number of frequency bands and types of noise did not have much effect on tone shape and tone height identification when the tones were presented in the original signal in quiet and in noise, as indicated by similar accuracy rates and error patterns in these conditions (Tables 12, 13, rows 1–27).

Taken together the results showed that tone heights were more difficult to discriminate even in original signals in quiet. Noise had no significant effect on the identification of tone heights in the original signal, but had a negative effect on the discrimination of differences in the rising slopes, resulting in slightly more confusion between T2 (HR) and T5 (LR) in original signals in noise. Number of frequency bands and noise types had little effect on tone shape or tone height identification in the original signal.

### Identification of tone heights and tone shapes in the temporal cues in quiet and in noise

When tones were presented in quiet, there was slightly more confusion of tone shapes in TFS than in the original signal, as there was more T2 (HR) being identified as T5 (LR) (Table 12, row 28), T4 (LF) being identified as T5 (LL) (Table 12, rows 35, 36), and T6 (LL) being identified as T5 (LR) (Table 13, rows 34–36) in TFS in quiet than in the original signal in quiet. Identification accuracies of tone heights in TFS in quiet also appeared to be slightly lower than in the original stimuli in quiet, as indicated by more T1 (HL) being perceived as T3 (ML) in TFS (Table 13, rows 28–30) than in the original signal in quiet (Table 13, rows 1–3). No clear effect of number of bands was found, as the accuracy rates were comparable in all frequency bands.

Thus, in the quiet condition, tone shape and tone height identification were slightly lower in TFS than in the original signal. Noise types and number of frequency bands had no strong effect on tone shape and tone height identification in TFS in quiet.

When the tones were presented in TFS in noise, there were slightly more tone shape errors than when the tones were presented in the original stimuli in noise. There were more T4 (LF) being identified as T5 (LR) in B16 (Table 12, rows 45 and 54) and more T3 (ML) and T6 (LL) being heard as T5 (LR) (Table 13, rows 44, 45, 51–53) than in the original stimuli in noise (Tables 12, 13, rows 10–27). No consistent effect of number of frequency bands or noise type on tone height and tone shape accuracies or error patterns was observed in TFS in noise. Thus, in noise condition, there were more tone shape errors in TFS in the two types of noise than in the original signal.

TE500 was a weak cue for identifying tone shapes in quiet and was an even worse cue for identifying tone heights in quiet. Table 12 (rows 55–63) shows that when the tones were presented in the quiet condition, over 50 and 95% of T2 (HR) and T4 (LF) were correctly identified. Though only 20–32% of T5 (LR) was correctly identified, most of the errors involved identifying T5 (LR) as T2 (HR), indicating correct identification of the rising contours but difficulty identifying small differences in the slope of the rising pitch in quiet. Yet, Table 13 (rows 55–63) shows that T1 (HL) and T3 (ML) in TE500 were most frequently heard as T4 (LF), indicating difficulties differentiating level and falling contours in quiet. TE500 in quiet was a poorer cue for tone heights than for tone shapes. There was few confusion among T1 (HL), T3 (ML), or T6 (LL). All the three level tones in TE500 in quiet were frequently heard as T4 (LF), a tone that was very different in tone height than T1 (HL) and T3 (ML) (See Wong and Chan, 2018).

When the tones were presented in TE500 in noise, there were substantially more tone shape and tone height errors than when the tones were presented in TE500 in quiet. Table 12 (rows 64–69, 73–78) and Table 13 (rows 64–81) show that T2 (HR), T5 (LR), T1 (HL), T3 (ML), and T6 (LL) were all most frequently heard as T4 (LF) tone.

TE50 was worse than TE500 for tone shape and tone height identification in quiet and in noise. Tables 12, 13 (rows 82–108) show that other than several exceptions in quiet for T2 (HR) and T5 (LR) (Table 12, rows 83, 84, 87), the six tones in TE500 were most frequently heard as T4 (LF), indicating difficulties identifying the tone shape and tone height differences in TE50 in quiet and in noise.

For both TE500 and TE50, error rates and patterns were similar across different number of frequency bands and types of noise, indicating little effect of noise type and frequency Bands in tone shape and tone height identification.

Altogether, tone height was more difficult to identify than tone shape in all acoustic cues, including the original signal in quiet. TFS was more effective than TE500 and TE50 for tone shape and tone height identification in quiet and in noise though there were more tone shape and tone height errors in TFS than in the original signal. TE500 and TE50 were not effective for tone shape or tone height identification, particularly when the cues were presented in noise. Tones presented in TE500 and TE50 were most frequently heard as T4 (LF) in TE500 and TE50 across all conditions in noise.

## Discussion

This is the first study to comprehensively examine the effects and interactions of temporal cues, background noises of different masking effects, and frequency bands on the identification of tone categories, tone shapes, and tone heights in a tone language (Cantonese) that uses both tone shapes and tone heights to distinguish lexical meanings.

The experimental procedures in this experiment involved asking participants to repeat tones they heard over headphones, and having a phonetically trained experimenter who was sitting next to the participant in front of the computer but did not hear the stimuli, to select the tone label of the produced tone for the participant. One limitation of this design is that tone judgment was not directly carried out by the listener and there is a possibility that the ratings of the experimenter did not truly reflect what the listeners perceived. The rationale for this study to adopt an indirect procedure in tone rating was based on the fact that some native Cantonese speakers may be able to perceive and produce tones but lack the metalinguistic skill to identify the tone labels because Cantonese tones are not specified in writing. In our previous research, we noted that non-phonetically trained participants may hear and produce one tone but label it as another. For example, they may produce T3 (ML) but identify it as T6 (LL), or vice versa. Labeling tones are more challenging in the task in this study because the six tones were presented in three different syllables in different acoustic cues and noises in which listeners might not be able to perceive the segmental structures of the stimuli correctly and come up with a lexical item. Future studies may provide training on tone distinction to participants prior to the tone identification experiment in order to collect direct judgment of tones from the listeners.

The possibility that the findings in this study were confounded by the inability of the participants to correctly perceive and produce the tones they heard can be ruled out for three reasons. First, the tones presented in the original signal in quiet were identified with accuracies and error patterns that were comparable to those reported in previous studies on Cantonese tone perception in adults. Second, only participants who were able to correctly perceive and imitate the six tones in the phone screening were recruited in the study. Third, any participant who scored 0% accuracy in any tones in the original stimuli in quiet was excluded.

The findings were not likely confounded by inaccurate tone ratings of the experimenters for several reasons. First, the experimenters were phonetically trained and pretested for Cantonese tone identification and production. Second, they attained high inter- and intra-judge reliability in rating the tones produced by the participants. Based on conventional interpretation, Kappa values of over 0.8 indicate almost perfect agreement (Landis and Koch, 1977). Third, the accuracy and error patterns across different acoustic cues and different conditions were highly consistent though the stimuli in different conditions were presented randomly in the same block and the experimenters did not hear the experimental stimuli. Forth, the accuracy rates and error patterns of the participants in the original stimuli in quiet were comparable to those in previous findings. Fifth, the participants could correct the experimenter if they thought the experimenter selected a wrong answer.

### Tone identification in original speech stimuli in quiet and in noise

When listening to the tones in the original stimuli under the quiet condition, most of the tones [T1 (HL), T2 (HR), T4 (LF) and T4 (LR)] were identified with very high (i.e., over 82%) accuracy. T3 (ML) and T6 (LL) were identified with lower accuracies of 70 and 42%, respectively. The error patterns revealed confusion between T3 (ML) and T6 (LL), suggesting that listeners had more difficulties identifying tone heights than tone shapes in the tones even in quiet.

The lower than perfect identification of Cantonese tones in isolated syllables in a quiet condition in adults has been reported previously (e.g., Lee et al., 2015; Wong et al., 2017; Wong and Chan, 2018). For example, Wong and Ng (2018) and Wong and Leung (2018) both reported that, among the six tones, T3 (ML) and T6 (LL) were identified with the lowest accuracy, with accuracy ranged from 65 to 77% (Wong and Leung, 2018; Wong and Ng, 2018). Confusion of this tone pair could be explained by similarities in tones shapes but relatively smaller pitch height differences between the two tones (Wong and Chan, 2018). The findings, therefore, suggested that identification of tone height is intrinsically more challenging even in quiet situations.

Noise had little effect on Cantonese tone perception in the original stimuli. When tones were presented in the original signal in 4 bands, identification accuracies of all tones in the two types of noises were comparable to those in quiet. When the original stimuli were presented in 8 and 16 bands in noise, only the rising tones [T2 (HR) and T5 (LR)] were perceived with lower accuracy in one of the two noises. The findings suggested that tone perception in the original signal is robust even when the tones were presented in the two noises at 0 dB SNR.

### Effectiveness of the temporal cues for tone identification

TFS was the most effective temporal cue for Cantonese tone identification in both quiet and noise, and tone identification in TFS was better in fewer frequency bands. Smith et al. (2002) also reported that TFS was relatively more important than temporal envelope cues for pitch perception. Based on tone identification in auditory chimera, Xu and Pfingst (2003), also found TFS a stronger cue than TE50 for Mandarin tone identification in quiet. Though T4 (LF), the falling contour, was more difficult to identify in TFS than in the two temporal envelope cues, when tones were presented in TFS in four bands, the identification accuracy of T4 (LF) reached high accuracies of 70% in quiet and above 80% in the two noises. Consistent with Kong and Zeng (2006), this study found that the TFS cue was less susceptible to noise. Identification accuracy of tones in TFS in the two noises was mostly comparable to the accuracy in the original stimuli in noise, particularly when the tones were presented in four bands. The two types of noise also did not have differential effect on tone identification accuracy in TFS, though SSN generated mostly energetic masking and 2MB induced mostly informational masking. The better tone perception with TFS with fewer number of frequency bands was consistent with previous findings by Smith et al. (2002). This could be attributed to more recovered envelope cues contained in TFS stimuli as well as heightened sensitivity of the auditory system to TFS with decreasing number of frequency bands (Ghitza, 2001).

Contrary to previous studies finding that temporal envelope cues were effective for Mandarin tone perception (Fu et al., 1998; Fu and Zeng, 2000), this study showed that neither TE500 nor TE50 were effective cues for Cantonese tone identification in quiet or in noise. Tones in TE500 and TE50 in quiet and in noise were identified with significantly lower accuracy than in TFS and ORI and many of the accuracy rates were close to or below chance level, except for T4 (LF). Though T4 (LF) was better identified in TE500 and TE50 than in TFS in quiet, the advantage was reduced in noise. Identification of T4 (LF) in TFS was as good as or better than in TE500 and TE50 in noise. Comparing the two temporal envelope cues, TE50 was worse than TE500 for Cantonese tone identification, supporting the findings in Fu and Zeng (2000) with Mandarin tones.

Yuan et al. (2009) was the only study comparing Cantonese tone identification in TE500 in quiet and in noise. They reported that when presented in TE500, Cantonese tones produced by a male speaker were identified with mean accuracies of about 92 and 90% in quiet and in SSN at 10 dB SNR, respectively (Figure 6 in Yuan et al., 2009). The accuracies were higher than those found in this study likely because of the differences in the aims and designs of the studies. This study focused on tone identification and asked participants to identify the tones in monosyllabic words that had the same segmental structures but differed in tones. Participants had to identify the auditorily presented word from 6 monosyllabic words minimally contrastive in the six tones. The tones in noise in this study were also presented at a lower SNR at 0 dB. Yuan et al. (2009) aimed at comparing word and tone identification in TE500. Participants listened to disyllabic words (e.g., [mei4 miu6] “subtle,” or [kei4 miu6] “miracle”) and were provided four words for selection (e.g., [mei4 miu6] “subtle,” [mei5 miu6] “wonderful,” [kei4 miu6] “miracle,” [mei5 maau6] “pretty”). Two of the word choices (e.g., [mei4 miu6] “subtle,” and [kei4 miu6] “miracle”) matched the disyllabic tone combination of the presented word. Participants' identification of either word was considered correct identification of the tones. The other two words differed from the presented word by the tone in the first or second syllable and may have the same or different segmental constructions as the presented word (e.g., [mei5 miu6] “wonderful,” and [mei5 maau6] “pretty”). Thus, participants in Yuan et al. (2009) had a 50% chance of selecting the right tone as opposed to the 17% chance rate in this study. Participants in Yuan et al. (2009) also had the coarticulation cue for tone identification [e.g., T6 (LL) after T4 (LF) is different from T6 (LL) after T5 (LR)]. In trials in which the presented word did not have a disyllabic word that differed only in tone from the presented word in the word choices, participants who could identify the segmental phonemes would be able to select the target word with the correct target tone without hearing the tones. For example, when [kei4 miu6] “miracle” was presented, if the participant heard the phoneme /k/, s/he would be able to select [kei4 miu6] “miracle” from the choices of [mei4 miu6] “subtle,” [mei5 miu6] “wonderful,” [kei4 miu6] “miracle,” [mei5 maau6] “pretty.”

### Perception of tone shapes and tone heights in different acoustic cues

Because lexical tones in different tonal languages may be contrasted by tone shapes, tone heights or both comparing tone shape and tone height identification accuracy in Cantonese in different temporal cues may shed light on tone identification in temporal cues in different tonal languages.

Tone heights appeared to be more difficult to identify than tone shapes. There was tone height confusion but no tone shape confusion in the original signal in quiet. Noise slightly deteriorated the perception of pitch slopes as indicated by more confusion between T2 (HR) and T5 (LR) when the tones were presented in the original signal in noise.

TFS was an effective cue for tone shape and tone height identification though not as good as in the original signal. There were more tone height [T1 (HL)-T3 (ML) confusion] and tone shape errors [T5 (LR)–T6 (LL) and T4 (LF)–T5 (LR)] in TFS than in the original signal in quiet and slightly more tone shape errors in TFS in noise than in the original signal in noise. Despite this, the accuracy rates of tones and error patterns were comparable in TFS and in the original signals in quiet and in noise, indicating that TFS was a strong cue for both tone shape and height identification.

TE500 and TE50 were weak acoustic cues for tone shape identification in quiet, and were even worse cues for tone heights identification in noise. In noise, listeners failed to correctly identify either tone shape or tone height when the tones were presented in TE500 or TE50. Most of the tones in TE500 and TE50 were heard as T4 (LF)in noise, and the three level tones were mostly identified at or below chance level in both quiet and noise.

### Effect of different maskers on tone identification in different acoustic cues

Most previous studies examining tone perception in noise used SSN and no study examined the effect of masker types on tone identification in different acoustic cues. Cheung (2015) examined the effect of four different maskers on the identification of Cantonese tone in original speech stimuli and reported that when the SNR was at 0 dB, noise type had no effect on tone accuracy, which was consistent with our finding that overall, no consistent difference was found for tone identification in 2MB or SSN. A new finding from this study was that even when the tones were presented in the three temporal cues, there was little difference in tone identification accuracy in the two types of noise. Cheung (2015) found that when the SNR was reduced to −6 dB, Cantonese tone perception was lower in SSN than in 2FB. This study did not examine the effect of SNR on tone perception in noise. Future studies will be needed to examine whether SNR differentially affects tone perception in different acoustic cues in different types of noise.

### Comparing mandarin and cantonese tone identification in different temporal cues

TFS appears to be a robust acoustic cue for both Mandarin (Xu and Pfingst, 2003) and Cantonese tone identification in quiet and in noise (Kong and Zeng, 2006), particularly when it is presented in fewer frequency bands.

Though TE500 and TE50 were significant cues for Mandarin tone identification (Fu et al., 1998; Fu and Zeng, 2000), they were ineffective for Cantonese tone perception. The discrepancies in the findings may be due to the fact that Mandarin tones are differentiated by pitch shapes only, while Cantonese tones are differentiated by both pitch shapes and pitch heights. Kuo et al. (2008) reported accuracy rates of 31, 48, 38, and 53% for the four Mandarin tones produced by a male speaker presented in TE50 in quiet. Fu and Zeng (2000) reported 36–78% tone identification accuracy in the four Mandarin tones presented in TE500 and 46–64% accuracy in TE50 in quiet. Fu et al. (1998) did not report tone accuracy by each Mandarin tone but found that, overall, the four tones were identified with 81 and 67% accuracy in TE500 and TE50, respectively, in quiet. Kong and Zeng (2006) was the only study that examined Mandarin tone production in TE50 and TE500 in quiet and in noise. The mean accuracy of the four tones in TE500 and TE50 was about 86 and 70% in quiet, respectively, when presented in 8 bands, and 60 and 52%, respectively, in SSN at 0 dB SNR. No information on the accuracy rates or errors of individual tones was provided. In our error analysis, contour tones in Cantonese such as T2 (HR) and T4 (LF) were identified with 52–98% accuracies in TE500 and 25–90% accuracies in TE50 in quiet, indicating that tone shapes could be differentiated in TE500 and TE50 in quiet. However, because Cantonese has other tones that differ by pitch heights and different degrees of the rising slop and because TE500 and TE50 cues were susceptible to noise, identification accuracies of most of the Cantonese tones in TE500 and TE50 in most conditions were low.

It appeared that the falling tone was very robust in TE500 and TE50 in Mandarin and Cantonese tone identification. Most previous studies reported that the Mandarin falling tone was identified with the highest accuracy in TE500 and TE50 (e.g., Fu and Zeng, 2000; Kuo et al., 2008) than the three other Mandarin tones in TE500 and TE50 in quiet. This study also found higher identification of the Cantonese falling tone than the other five tones in TE500 and TE50 in quiet and in noise. Error analyses showed that in TE500 and TE50, particularly when tones were presented in noise, most tones regardless of pitch shapes and pitch heights were most frequently heard as T4 (LF), which could explain why falling tone was identified with the highest accuracy. Possible reasons for the excessive T4 (LF) responses could be that TE500 and TE50 cues may elicit a percept of a falling pitch. Another reason could be that because T4 (LF) was the only tone they could hear in challenging situations, listeners may develop a response strategy and tend to select T4 (LF) when they had no clue what the presented tone was.

Previous studies examining the effect of number of frequency bands on Mandarin tone identification in temporal cues reported mixed results. Fu et al. (1998) found no effect of frequency bands on tone perception in TE500 and TE50 when the tones were presented in one to four bands in quiet. Xu et al. (2002) reported that in quiet, better tone identification accuracy was found in TE500 with fewer frequency bands or in TE50 with more frequency bands, suggesting that TE500 was more effective in fewer frequency bands while TE50 was more effective in more frequency bands. Kong and Zeng (2006) found that in quiet situation, tone identification accuracy in TE500 in one band was better than TE50 in 8 bands, indicating that tone identification accuracy in TE500 in one band was better than TE50 in more bands. However, the pattern was reversed when the Mandarin tones were presented in noise. This study found better tone identification in TFS with fewer frequency bands in quiet and in noise but did not find any effect of bands on the two temporal envelope cues in quiet or in noise, except that tone identification in TE50 in quiet was better in 16 bands than in 4 bands. The discrepancies in the findings in this study and previous studies on Mandarin can be attributed to the differences in the experimental design. First, none of the previous studies examined the effect of frequency bands on tone identification in TFS. Second, previous Mandarin studies compared tone accuracies in up to 12 frequency bands while this study examined tone perception accuracy in up to 16 bands. Third, except Kong and Zeng (2006), the other studies did not compare tone perception in noise. It is likely that when all these factors are taken into consideration, little effect of bands was found for the two temporal envelope cues. Another possible reason for the differences in the findings could be that this study examined Cantonese tones while other studies examined Mandarin tones. In any case, because most of the Cantonese tones in TE500 and TE50 were identified with low accuracies (mostly below 30% and some below chance level) in quiet and noise across all frequency bands, TE500 and TE50 were ineffective cues for Cantonese tone identification regardless of number of frequency bands. Yet, for TFS, the best tone identification can be achieved in fewer frequency bands.

## Implications

Discrepant findings on tone identification in the temporal envelope cues in Mandarin and Cantonese indicate that findings on Mandarin tones are not applicable to Cantonese tones, since tone identification accuracy is affected by the complexity of the tonal system in terms of number of contrastive tones and the nature of the tonal contrasts. Mandarin has a relative simple tonal system with only 4 tones and the tones are only contrasted by tone shapes. On the other hand, Cantonese has six tones and the tones are contrasted by both tone shapes and tone heights. Due to the differences in the chance level, higher tone identification accuracies in the temporal envelope cues were found in Mandarin than in Cantonese. Given that Cantonese is contrastive in both pitch heights and shapes, it is predicted that findings on Cantonese tones are more applicable to other tonal languages and music appreciation. Because TFS is robust for both tone shape and tone height identification, it would likely be a reliable cue for tone perception across different tonal languages.

Current cochlear implants mostly deliver temporal envelope cues to users (Kong and Zeng, 2006). Findings in this study show that they would not be sufficient for tone identification for Cantonese speaker. Thus, Cantonese cochlear implant users would encounter greater difficulty in communication than Mandarin-speaking users and non-tonal language users.

Future cochlear implant processors may target at providing TFS cues in fewer frequency bands for better tone perception and music appreciation given that TFS is a strong cue for both tone shape and tone height identification, tone identification is better in fewer number of bands, and TFS is minimally affected by noise and noise types.

## Conclusions

Across all different acoustic cues, including the original acoustic signal, identification of pitch height is more difficult than identification of pitch shape in both quiet and noise. Among the three temporal cues, TFS is the most effective cue for the identification of tone shapes, tone heights and tone categories in Cantonese in quiet and in noise, particularly when tones are presented in fewer frequency bands. TE500 and TE50 are not effective for Cantonese tone perception in quiet or in noise though they are the strongest cue for the identification of T4 (LF). Types of masking do not significantly affect tone identification of the temporal cues. Cantonese tone identification accuracies in 2MB and in SSN are mostly comparable. Discrepancies in the findings of studies that examined the effects of temporal cues on the perception of Mandarin and Cantonese tones indicate that findings on Mandarin tones may not be generalizable to Cantonese tones. Future cochlear implant processors may take into consideration the characteristics of these acoustic cues for pitch perception when designing future cochlear implants for tone users.

## Author contributions

PW designed the experiment, analyzed the data, and prepared the manuscript. SC collected the data. PW and SC performed analyses and drafted an earlier version of the work. FC generated part of the synthetic stimuli.

### Conflict of interest statement

The authors declare that the research was conducted in the absence of any commercial or financial relationships that could be construed as a potential conflict of interest.

## References

[B1] Barry J. G. Blamey P. J. Martin L. F. A. Lee K. Y. S. Tang T. Ming Y. Y. (2002). Tone discrimination in Cantonese-speaking children using a cochlear implant. Clin. Linguist. Phon. 16, 79–99. 10.1080/02699200110109802 11987495

[B2] Bauer R. S. Cheung K. Cheung C. (2003). Variation and merger of the rising tones in Hong Kong Cantonese. Lang. Variat. Change 15, 211–225. 10.10170/S0954394503152039

[B3] Cheung M. S. (2015). Cantonese tone Perception in Noise in Young and Aged Healthy Adults. Unpublished dissertation, The Univerisity of Hong Kong.

[B4] Freyman R. L. Balakrishnan U. Helfer K. S. (2004). Effect of number of masking talkers andauditory priming on informational masking in speech recognition. J. Acoust. Soc. Am. 115, 2246–2256. 10.1121/1.689343 15139635

[B5] Fu Q.-J. Zeng F.-G. (2000). Identification of temporal envelope cues in Chinese tone recognition. Asia Pac. J. Speech Lang. Hear. 5, 45–57. 10.1179/136132800807547582

[B6] Fu Q.-J. Zeng F.-G. Shannon R. V. Soli S. D. (1998). Importance of tonal envelope cues in Chinese speech recognition. J. Acoust. Soc. Am. 104, 505–510. 10.1121/1.423251 9670541

[B7] Ghitza O. (2001). On the upper cutoff frequency of the auditory critical-band envelope detectors in the context of speech perception. J. Acoust. Soc. Am. 110, 1628–1640. 10.1121/1.1396325 11572372

[B8] Greenwood D. D. (1990). A cochlear frequency-position function for several species-29 years later. J. Acoust. Soc. Am. 87, 2592–2605. 10.1121/1.399052 2373794

[B9] Kong Y.-Y. Zeng F.-G. (2006). Temporal and spectral cues in Mandarin tone recognition. J. Acoust. Soc. Am. 120, 2830–2840. 10.1121/1.2346009 17139741

[B10] Kuo Y.-C. Rosen S. Faulkner A. (2008). Acoustic cues to tonal contrasts in Mandarin:implications for cochlear implants. J. Acoust. Soc. Am. 123, 2815–2824. 10.1121/1.2896755 18529197

[B11] Landis J. R. Koch G. G. (1977). The measurement of observer agreement for categorical data. Biometrics 33, 159–174. 10.2307/2529310 843571

[B12] Lecumberri M. L. G. Cooke M. (2006). Effect of masker type on native and non-native consonant perception in noise. J. Acoust. Soc. Am. 119, 2445–2454. 10.1121/1.2180210 16642857

[B13] Lee K. Y. S. Chan K. T. Y. Lam J. H. S. van Hasselt C. A. Tong M. C. F. (2015). Lexical tone perception in native speakers of Cantonese. Int. J. Speech Lang. Pathol. 17, 53–62. 10.3109/17549507.2014.898096 24780063

[B14] Loizou P. C. (1998). Mimicking the human ear. Signal Process. Mag. IEEE 15, 101–130. 10.1109/79.708543

[B15] Mattys S. L. Brooks J. Cooke M. (2009). Recognizing speech under a processing load: dissociating energetic from informational factors. Cogn. Psychol. 59, 203–243. 10.1016/j.cogpsych.2009.04.001 19423089

[B16] Moore B. C. J. Peters R. W. (1992). Pitch discrimination and phase sensitivity in young and elderly subjects and its relationship to frequency selectivity. J. Acoust. Soc. Am. 91, 2881–2893. 10.1121/1.402925 1629481

[B17] Oxenham A. J. (2008). Pitch perception and auditory stream segregation: implications for hearing loss and cochlear implants. Trends Amplif. 12, 316–331. 10.1177/1084713808325881 18974203 PMC2901529

[B18] Plag I. Kunter G. Schramm M. (2011). Acoustic correlates of primary and secondary stress in North American English. J. Phon. 39, 362–374. 10.1016/j.wocn.2011.03.004

[B19] Rosen S. (1992). Temporal information in speech: acoustic, auditory and linguistic aspects. Philos. Transac. R. Soc. Lond. B Biol. Sci. 336, 367–373. 1354376 10.1098/rstb.1992.0070

[B20] Shannon R. V. Zeng F. G. Kamath V. Wygonski J. Ekelid M. (1995). Speech recognition with primarily temporal cues. Science 270, 303–304. 10.1126/science.270.5234.303 7569981

[B21] Smith Z. M. Delgutte B. Oxenham A. J. (2002). Chimaeric sounds reveal dichotomies in auditory perception. Nature 416, 87–90. 10.1038/416087a 11882898 PMC2268248

[B22] Wong L. L. Soli S. D. (2005). Development of the Cantonese hearing in noise test (CHINT). Ear. Hear. 26, 276–289. 10.1097/00003446-200506000-00004 15937409

[B23] Wong P. Chan H.-Y. (2018). Acoustic characteristics of highly distinguishable Cantonese entering and non-entering tones. J. Acoust. Soc. Am. 143, 765–779. 10.1121/1.5021251 29495691

[B24] Wong P. Fu W.-M. Cheung E. Y.-L. (2017). Cantonese-speaking children do not acquire tone perception before tone production – A perceptual and acoustic study of three-year-olds' monosyllabic tones. Front. Psychol. 8:1450. 10.3389/fpsyg.2017.01450 28900404 PMC5581918

[B25] Wong P. Leung C. T. (2018). Suprasegmental features are not acquired early: perception and production of monosyllabic Cantonese lexical tones in four- to six-year-old children. J. Speech Lang. Hear. Res. 61, 1070–1085. 10.1044/2018_JSLHR-S-17-0288 29710319

[B26] Wong P. Ng K. W. S. (2018). Testing the hyper-articulation and prosodic hypotheses of child directed speech: insights from the perceptual and acoustic characteristics of child-directed Cantonese tones. J. Speech Lang. Hear. Res. 61, 1907–1925. 10.1044/2018_JSLHR-S-17-0375 30073296

[B27] Xu L. Pfingst B. E. (2003). Relative importance of temporal envelope and fine structure in lexical-tone perception (L). J. Acoust. Soc. Am. 114, 3024–3027. 10.1121/1.1623786 14714781 PMC1283139

[B28] Xu L. Tsai Y. Pfingst B. E. (2002). Features of stimulation affecting tonal-speech perception:implications for cochlear prostheses. J. Acoust. Soc. Am. 112, 247–258. 10.1121/1.1487843 12141350 PMC1414789

[B29] Yuan M. Lee T. Yuen K. C. P. Soli S. D. van Hasselt C. A. Tong M. C. F. (2009). Cantonese tone recognition with enhanced temporal periodicity cues. J. Acoust. Soc. Am. 126:327. 10.1121/1.3117447 19603889

[B30] Yuen K. C. P. Yuan M. Lee T. Soli S. Tong M. C. F. van Hasselt C. A. (2007). Frequency specific temporal envelope and periodicity components for lexical tone identification in Cantonese. Ear. Hear. 28, 107S–113S. 10.1097/AUD.0b013e31803153ac 17496660

